# High-Resolution Crystal Structures of Protein Helices Reconciled with Three-Centered Hydrogen Bonds and Multipole Electrostatics

**DOI:** 10.1371/journal.pone.0123146

**Published:** 2015-04-20

**Authors:** Daniel J. Kuster, Chengyu Liu, Zheng Fang, Jay W. Ponder, Garland R. Marshall

**Affiliations:** 1 Department of Biomedical Engineering, Washington University, St. Louis, MO, United States of America; 2 Department of Chemistry, Washington University, St. Louis, MO, United States of America; 3 Department of Biochemistry and Molecular Biophysics, Washington University, St. Louis, MO, United States of America; Wake Forest University, UNITED STATES

## Abstract

Theoretical and experimental evidence for non-linear hydrogen bonds in protein helices is ubiquitous. In particular, amide three-centered hydrogen bonds are common features of helices in high-resolution crystal structures of proteins. These high-resolution structures (1.0 to 1.5 Å nominal crystallographic resolution) position backbone atoms without significant bias from modeling constraints and identify Φ = -62°, ψ = -43 as the consensus backbone torsional angles of protein helices. These torsional angles preserve the atomic positions of α-β carbons of the classic Pauling α-helix while allowing the amide carbonyls to form bifurcated hydrogen bonds as first suggested by Némethy et al. in 1967. Molecular dynamics simulations of a capped 12-residue oligoalanine in water with AMOEBA (Atomic Multipole Optimized Energetics for Biomolecular Applications), a second-generation force field that includes multipole electrostatics and polarizability, reproduces the experimentally observed high-resolution helical conformation and correctly reorients the amide-bond carbonyls into bifurcated hydrogen bonds. This simple modification of backbone torsional angles reconciles experimental and theoretical views to provide a unified view of amide three-centered hydrogen bonds as crucial components of protein helices. The reason why they have been overlooked by structural biologists depends on the small crankshaft-like changes in orientation of the amide bond that allows maintenance of the overall helical parameters (helix pitch (**p**) and residues per turn (**n**)). The Pauling 3.6_13_ α-helix fits the high-resolution experimental data with the minor exception of the amide-carbonyl electron density, but the previously associated backbone torsional angles (Φ, Ψ) needed slight modification to be reconciled with three-atom centered H-bonds and multipole electrostatics. Thus, a new standard helix, the 3.6_13/10_-, Némethy- or N-helix, is proposed. Due to the use of constraints from monopole force fields and assumed secondary structures used in low-resolution refinement of electron density of proteins, such structures in the PDB often show linear hydrogen bonding.

## Introduction—Fundamental Assumptions of the Classic α- and 3_10_-Helical Models

**Fig 1:**
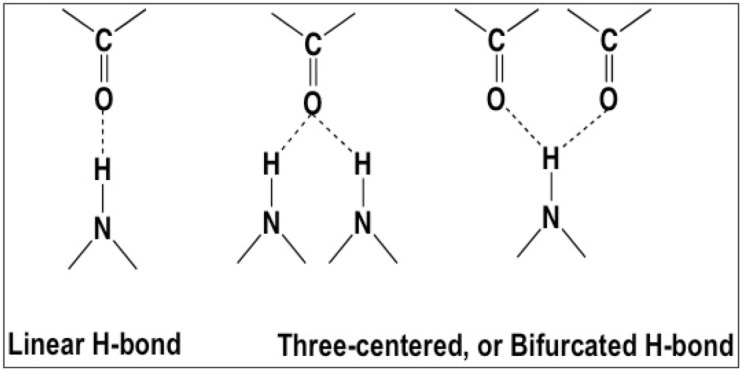
Hydrogen-bonding alternatives; linear H-bond assumed by the Pauling/Donohue groups; three-centered hydrogen bonds to carbonyl oxygens that dominate crystal structures of small organic molecules [6].

The α-helix model is the result of powerful deductions about the chemistry of amide bonds and peptides by Pauling [1]. First, amide bonds were planar due to resonance, a fact not appreciated by others in the field (see Eisenberg [2]); second, intramolecular hydrogen bonding needed to be optimized; and third, there was no reason to restrict a model to an integral number of residues per turn. In 1951, Pauling, Corey and Branson [1] applied these constraints in the context of regular protein sequences to yield the α-helix, which originally had 3.7 residues-per-turn [3] and 13 atoms in a hydrogen-bonded ring (i.e., α-helix = 3.7_13_-helix). Two critical assumptions were: the concept of linear groups (i.e., a series of residues all having identical conformations), and the use of single, linear hydrogen bonds (**Fig 1**) to fold the sequence in space by imposing a distance constraint between two non-consecutive amino acids in space. In one of the subsequent nine papers Pauling and Corey published in 1951, they suggested, “The number of residues per turn is fixed primarily by the bond angle at the α carbon atom; it varies from 3.60 for bond angle 108.9° to 3.67 for bond angle 110.8°”. The backbone torsional angles (Φ = -57°, Ψ = -47°) associated with the α-helix (3.6_13_-helix) became official in the article by the IUPAC-IUB Commission on Biochemical Nomenclature published in 1970 [4]. These values were based on experimental fitting of parameters to diffraction data for poly-L-alanine in 1967 by Arnott and Dover [5].

**Fig 2:**
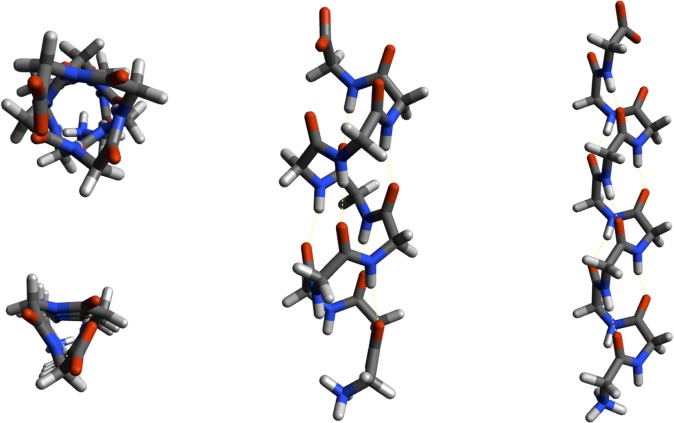
Schematic stick-figure diagrams of α-helix (top left and middle) and 3_10_-helix (bottom left and right) helices.

**Fig 3:**
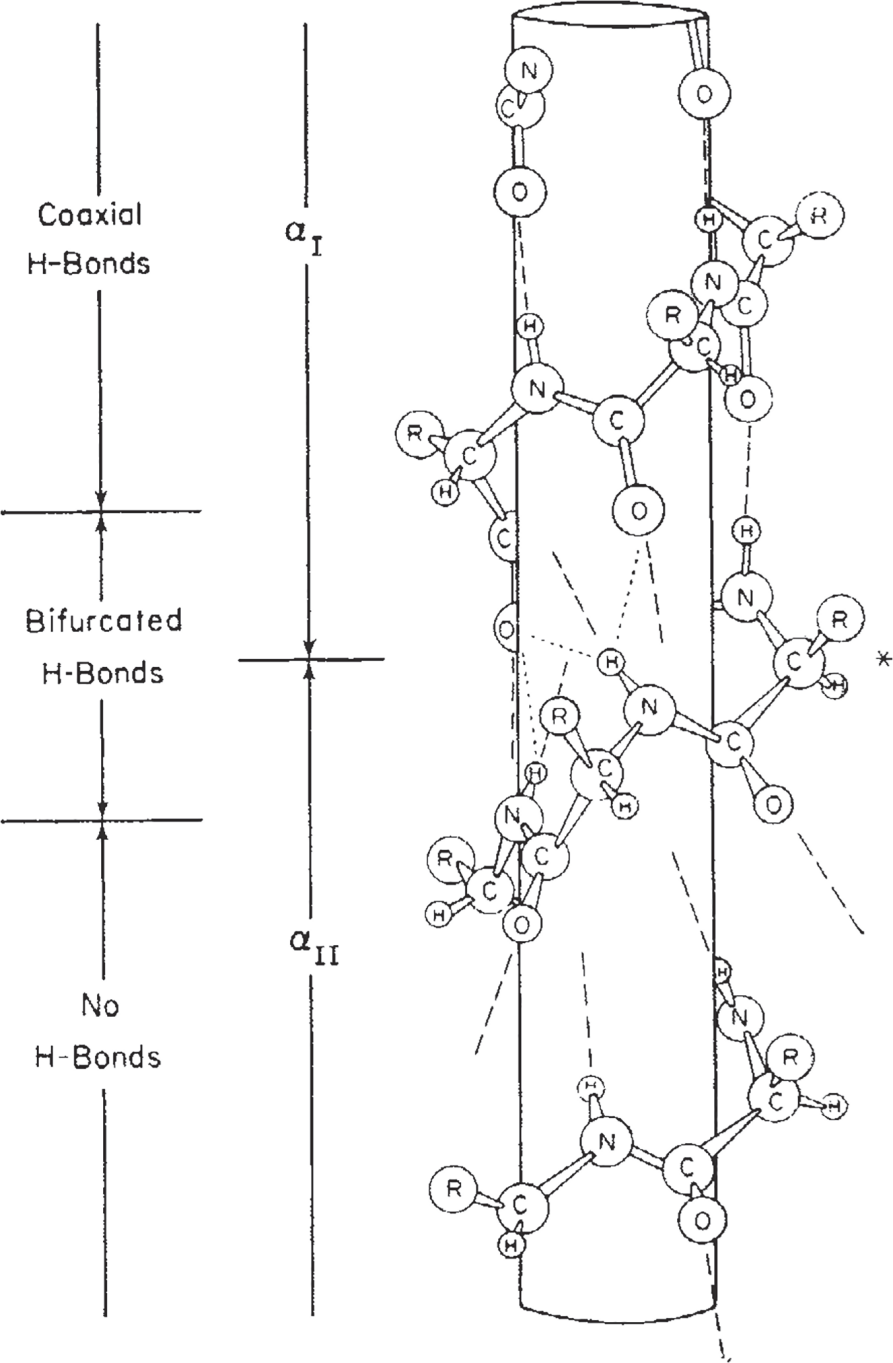
Reproduction of Fig 1 from Némethy et al. [9] showing an intermediate N-helix (3.6_13/10_) with the helical parameters of the classical α-helix and bifurcated amide H-bonds (used with permission).

In 1953, Donohue applied similar constraints with slightly different parameters to arrive at an alternative helical configuration (**Fig 2**), one with a rise of 3 residues-per-turn and 10 atoms (3_10_-helix) in the hydrogen-bonded ring [7]. Where the α-helix places residues *i* and *i+4* with a hydrogen bond, the 3_10_-helix is a longer and more tightly wound configuration with hydrogen bonds between residues *i* and *i+3* (**Fig 2**). Némethy et al. analyzed the equations derived by Miyazawa [8] regarding helical parameters (helix pitch (**p**) and residues per turn (**n**)) and proposed the α_II_-helix in 1967 that exactly fit the helical parameters of the Pauling α-helix, but lacked amide hydrogen bonds [9]. As the importance of amide H-bonds and three-centered hydrogen bonding (**Fig 1**) was just beginning to be appreciated [10], the α_II_-helix disappeared from the literature. In the Némethy et al paper [9], however, an intermediate helix between the α-helix and the α_II_-helix was illustrated (**Fig 3**) with bifurcated hydrogen bonds and the same helical pitch and residues-per-turn as the Pauling α-helix. These are exactly the properties observed for helices in high-resolution crystal structures from the Protein Data Bank (PDB) [11] that we refer to as Némethy-, N- or 3.6_13/10_-helices in this paper. The crucial insight by Némethy et al. was recognition that helical parameters are not tightly coupled to backbone torsional angles.

The canonical α-helix and 3_10_-helix, which are the result of logically applying simple but powerful assumptions, laid the foundation for structural biology and dominated the interpretation of experimental data for over half a century. Whereas Pauling, Donahue and Némethy had access to very little structural data, today an abundance of structural data is available [12]. Armed with improved experimental data from over 100,000 protein structures, it is possible to revisit assumptions and refine parameters for protein helices without imposing restrictive assumptions.

### Classical assumptions vs. high-resolution data

In the intervening half-century, detailed understanding of molecular interactions as well as the surfeit of precise experimental data has led to concern about the early assumptions that linear hydrogen bonds are optimal electrostatic interactions between amides, and that linear groups apply to proteins. Moreover, early crystallographic data were often limited in resolution and employed external force field and/or secondary structural constraints during refinement; in turn, force fields were validated on crystallographic data, a “circular reference” potentially leading to propagation of errors. More sophisticated methods developed for small-molecule structures exemplified by REFMAC [13] or SHELXL [14] only began to take root for structural refinement of proteins in the middle of the 1990s. MD simulations with a requisite force field (monopole electrostatics that reinforce linear hydrogen bonding) were introduced in 1991 by Axel Brunger and used widely [15]. It is, in fact, significant improvements in crystallographic technology and refinement in the last two decades that have provided the high-resolution protein structures on which this research is based. Nevertheless, REFMAC5 provides low-resolution refinement tools that utilize secondary-structural constraints when the experimental observations are of poor quality [13,16]. Systematic changes in helical torsional angles from PDB structures when binned by crystallographic resolution imply systematic error in data derived from low-resolution electron diffraction data.

### Linear hydrogen bonds

Both Pauling et al. [1] and Donahue [7] assumed a linear hydrogen bond between the carbonyl oxygen and the amide hydrogen of amide bonds, in order to place geometrical constraints on their helical models (**Fig 2**). Pauling et al. “assumed that each nitrogen atom forms a hydrogen bond with an oxygen atom of another residue, with the nitrogen-oxygen distance equal to 2.72 Å, and that the vector from the nitrogen atom to the hydrogen-bonded oxygen atom lies not more than 30° from the N-H direction.” The 3.7_13_-helix described had the angle between the N-H vector and N-O vector of ~10° [1]. This assumption has been computationally perpetuated by the use of monopole electrostatics in first-generation molecular mechanics. Although linear hydrogen bonds were a reasonable first-order approximation in 1951, more accurate treatment of electrostatics supports three-centered hydrogen bonds as seen in high-resolution crystal structures.

It has been known for some time from crystal structures of small molecules, that carbonyl oxygens do not form linear hydrogen bonds [6] as the total electron density due to the lone pairs is not geometrically positioned [17] to stabilize such interactions. For example, the hydrogen-bonded interactions seen experimentally in water clusters cannot be adequately reproduced by monopole electrostatics; in that case, the angle is approximately 57°, far from linear [18]. In fact, accurate electrostatic potentials surrounding small molecules (such as water) cannot be faithfully represented by monopole electrostatics; the addition of dipole and quadrupole terms is required [19–22]. The force field models (CHARMM, AMBER, OPLS-AA, etc.) commonly used in molecular modeling of proteins [23] utilize monopole electrostatics that produce linear hydrogen bonds between amide hydrogens and carbonyl oxygens. This can be demonstrated by minimizing the formula (Jeans’ formula) expressing the potential energy V of interaction between two dipoles as shown in Eq (1). Taking the derivative and setting cosθ_12_ to zero gives the minimum of the angles between the dipoles at 0^°^ or 180^°^ (i.e., a linear hydrogen bond).

$$V\left(r\right)\;=\;-\mu 1\cdot \mu 2\;\cdot (\cos\theta_{12}-3\cos\theta_{1}\cdot \cos\theta_{2})$$ 1

where μ = dipoles vectors, r = distance between the dipoles, θ = angle between the dipoles.

Hydrogen bonds are dominated by electrostatics, consequently, energy minimization or molecular dynamics (MD) simulations using force fields with monopole electrostatics will overemphasize helices with linear hydrogen bonds. Note that Pauling and Donahue did not choose linear hydrogen bonds (and thereby the canonical α- and 3_10_-helical configurations) with the belief that hydrogen bonds must always be linear in helices, but rather because Coulombic electrostatics could be solved and interpreted analytically without computers, and because these simple potentials were adequate to explain the very limited amount of experimental data available at the time. Others have questioned probable helical bias in force fields commonly used by molecular mechanics [23,24]. As these first-generation force fields have been often used in analysis of experimental X-ray diffraction and NMR structures [15], there is valid concern regarding helical bias in the primary source of protein structural data, the PDB [11]. Similar observations regarding the discrepancies between theoretical/experimental observations and the results of molecular modeling of side-chain hydrogen bonds were made by Morozov et al. [25].

Recently, the limitation of monopole electrostatics has led to the development of second-generation force fields (such as AMOEBA [15,26]), which include both multipole electrostatics and polarizability for proteins and nucleic acids. The results obtained with AMOEBA are under scrutiny to validate the ability of improved electrostatics to predict experimentally measured thermodynamic quantities [27]. The relative orientation of water molecules in an AMOEBA simulation of water clusters matches that calculated by high-level *ab initio* quantum mechanical models, in contrast to the linear hydrogen bonding seen with monopole force fields [18]. AMOEBA has been evaluated for its ability to reproduce experimental electron density in peptide crystals [28] with only minor adjustments needed to get quantitative fits. Fenn et al. have shown recently that AMOEBA can correctly orient water networks and catalytically relevant hydrogens in proteins [29]. In a recent comparison of the ability of MD simulations to predict experimentally observed NOEs (nuclear Overhauser effects), AMOEBA was vastly superior to the three monopole force fields commonly used [30]. Thus, the computational tools to employ accurate physical approximations for electrostatics while exploring the effects of structural motifs on the dynamic behavior of proteins are now available [31].

### Linear groups assume that all amino acid residues in a helix have identical conformations

Another simplifying assumption employed by Pauling and Donahue was the presence of “linear groups”, defined as sequences of three consecutive amino acid residues all having the same [ϕ,ψ]-angles ±10^°^. The linear group assumption has not been supported by structural studies, and where they have been validated, the definition of “linear group” has been permissive enough to accommodate ensembles of similar-but-not-identical configurations. For example, according to a 2009 survey of high-resolution crystal structures of proteins by Hollingsworth et al. [32], only three distinct linear groups were found in 135 proteins. The most populated was a right-handed helical conformation dominated by the α-helix, but also included the 3_10_-helix and mixed α/3_10_-helices. The other two linear groups detected were: a diverse group of extended conformations that are dominated by residues occurring in β-strands (in both parallel and antiparallel β-sheets), and a set of left-handed spiral conformations in the PII area.

### Implications of crystallographic resolution

**Fig 4:**
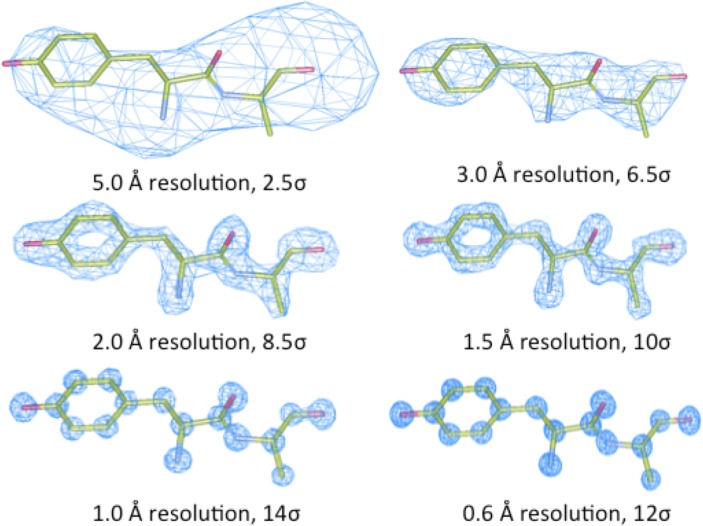
The appearance of electron density as a function of the nominal resolution of the experimental crystallographic data, (adapted from similar figure for the N-terminal fragment (Lys1—Val2—Phe3) of triclinic lysozyme (PDB: 2vb1) from Wlodawer et al. [33] for which permission to reproduce by the journal was not granted).

Fortunately, the quality of protein crystal structures has improved dramatically over the past several decades. Early protein crystals diffracted poorly, and data collection was generally limited to a 2.5 Å resolution, or greater. Wlodower et al. illustrated the impact of nominal resolution on the electron density map from which structural models are derived [33] (**Fig 4**shows a similar comparison based on crambin 3NIR). Whereas the highest resolution of 0.65 Å in the lysozyme structure employed data from 184,676 reflections for the electron-density map calculation, only 415 reflections were included at 5 Å resolution. Note that while higher-resolution maps provide enough geometric detail to unambiguously determine the amide plane and C = O vectors, there are multiple ways one could build a molecular model into density maps of poorer resolution [33]. X-ray crystallography is capable of localizing heavy atoms with astounding accuracy, so how is it possible for errors in helical populations to go undetected in so many crystallographic experiments? Interestingly, there is a straightforward two-part explanation: (i) hydrogens are less visible to X-ray diffraction due to their very small electronic cross-section; and (ii) the crystallographically determined positions of the heavy atoms, Cα-C-N-Cα, of the peptide backbone specify a unique helical configuration, i.e. helical parameters, the number of residues per turn and number of atoms in a hydrogen-bonding ring that was originally determined by fiber diffraction. The backbone torsional parameters (Φ, ψ) for helical models having the same helical parameters, however, are determined by the orientation of the carbonyl C = O vector which have limited, but significant, degrees of freedom for the same helical parameters based on the insight of Némethy et al. [9].

The 3_10_-helix is elongated compared to the α-helix (**Fig 2**), a consequence of its tighter helical twist that is imposed by *i→i+3* vs. *i→i+4* hydrogen bonding pattern for the α-helix. As the 3_10_-helix is longer than the α-helix, one might have expected changes in helical parameters (i.e., different number of residues-per-turn, helical pitch, position of Cαs and orientation of Cα-Cβ vectors) as the backbone torsional angles moved between those of the classical α-helix towards that of the 3_10_-helix. However, the torsional parameters for ϕ and ψ can change significantly without perturbing Cα atomic positions as pointed out by Némethy et al. in 1967 [9]. Crankshaft motions of torsional angles reorient the peptide plane and amide carbonyl without changing positions of Cα–Cβ atoms, i.e. the helical parameters. Whereas small changes in backbone torsion angles change the orientation of the amide bond to allow amide three-centered hydrogen bonds to optimize electrostatic interactions, unless the resolution is sufficient to resolve the position of the carbonyl oxygen within the helical cylinder, there is little difference during refinement of a molecular model to fit experimental electron density.

Determining an accurate position and orientation of the carbonyl oxygen of the peptide bonds is difficult (**Fig 4**) until the resolution is less than approximately 2.0 Å. Because hydrogens are less-well resolved by X-ray scattering in protein crystallography, the H-N amide orientation is effectively invisible, and backbone torsional [ϕ, ψ] parameters depend on the detailed orientation of the modeled carbonyl C = O vectors. For data gathered at poorer resolutions, fine-grained details were irrelevant, and the classic α-helical cylinder, refined with the assistance of monopole force fields, was assumed to fit the ambiguous geometric constraints. Thus, models built from low-resolution data (>2.0 Å) are susceptible to introduction of bias from modeling assumptions, and (in the absence of experimental constraints) might be expected to model classic linear groups. Models built from high-resolution data (<2.0 Å) are sufficiently constrained by experimental factors to model the detailed configurations of helical backbones without imposing such limiting assumptions.

### High-resolution models have less bias than poorer-resolution models

Careful analyses of high-resolution (<2.0 Å) protein crystallographic models have, for some time, revealed discrepancies between models supported by accurate experimental data and the classical helical models. More than thirty years ago, Blundell et al. [34,35] recognized how the solvent-shielded interior of a helix tended to have longer and less linear hydrogen bonds, “. . .recent high-resolution (1.7 − 2.0 Å) diffraction studies have shown that the parameters described by Pauling et al and later by Perutz [35] and by Arnott and Wonacott [36] are not a good description of the α-helices in globular proteins, where the mean values of ϕ, ψ are usually close to -63 degrees, -42 degrees. . .and arose as a mean of two significantly different classes in amphipathic helices depending on whether the peptide carbonyl oxygen is hydrogen bonded to a solvent or polar side-chain atom. The hydrogen bonds made by the hydrophilic carbonyls to the NH groups within helices are longer and less linear than those involving hydrophobic carbonyls.” [34]

## Results and Discussion

### Analysis of protein helices binned by nominal crystallographic resolution

**Fig 5:**
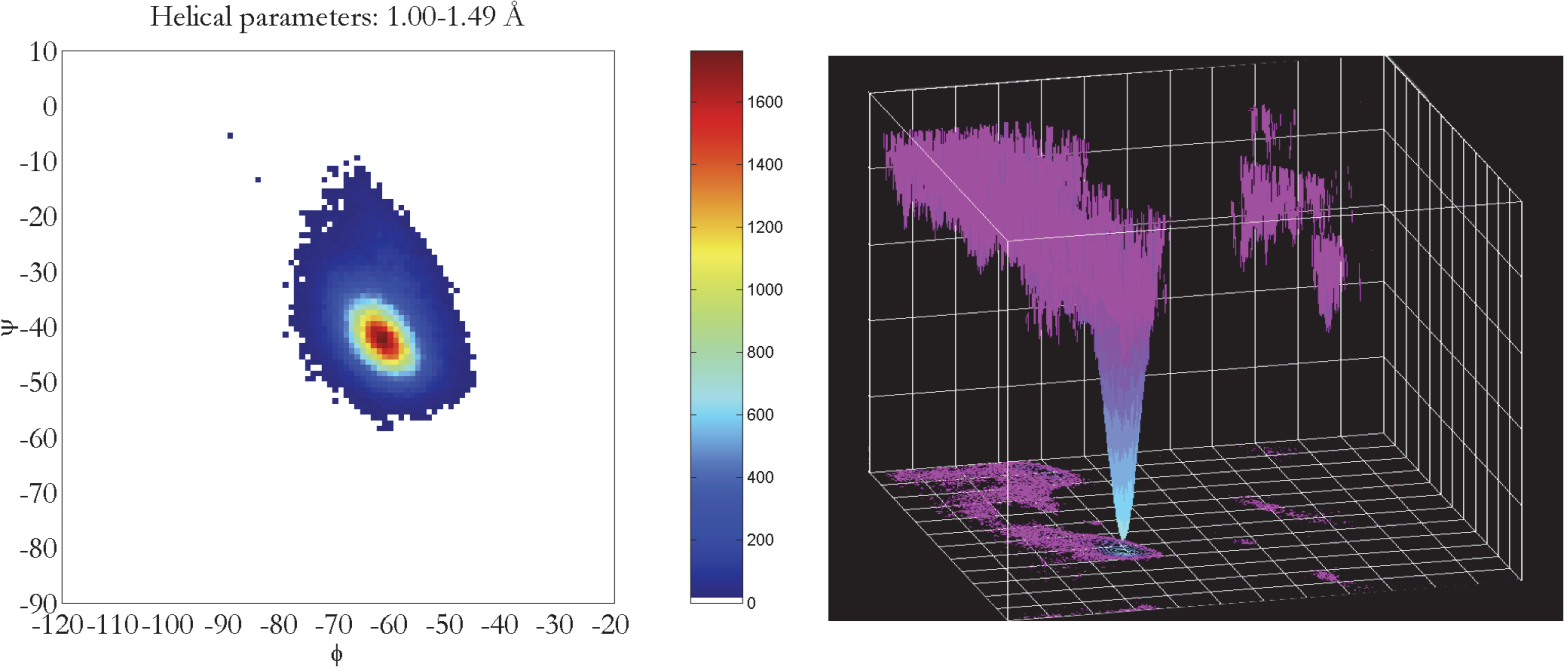
Left: Distribution of helical backbone torsion angles (Φ, Ψ) for 1.0–1.5 Å nominal resolution of 3,462 protein structures from the PDB. Right: Conversion of density of high-resolution helices to a relative energy scale by Boltzmann-weighting the distribution.

**Fig 6:**
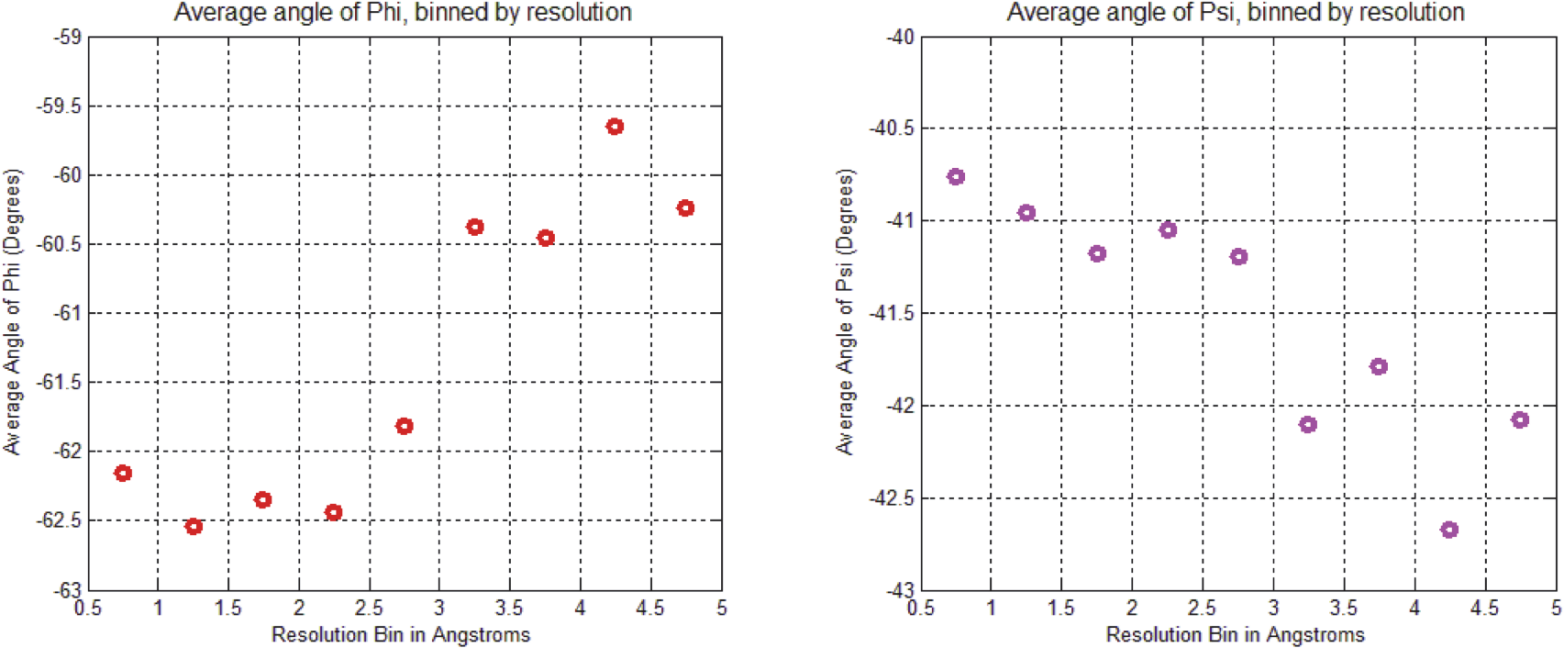
Average values of Φ (Phi) and Ψ (Psi) torsion angles of helical residues in PDB binned by nominal crystallographic resolution. This compensatory change in Φ, Ψ is due to a “crankshaft” motion [39] of the amide bond that maintains the relative positions of the α-carbons of the peptide backbone despite significant deviations in Φ, Ψ torsions.

Analysis of all experimental structural models of helices in proteins in the Protein Databank (**Table 1**) exposed subtle inconsistencies in the torsional angle (Ψ, ϕ) parameters, when comparing high-resolution data against the classic α/3_10_-helical models. At high resolution, where the experimental electron density accurately constrained helical conformations without the need for any structural assumptions or force-field constraints [33], the backbone torsional angles populate a smooth distribution of helical conformations with a single maximum (**Fig 5**) at ϕ = -62°, Ψ = -43° backbone torsion angles rather than the anticipated bimodal distribution of α- and 3_10_-helices [37]. Hovmoller et al. had previously analyzed a non-redundant set of 1042 protein subunits from the PDB (January 3, 2002) with regard to secondary structure. The proteins were all determined by X-ray diffraction to a resolution of 2.0 Å or higher, refined to R ≤ 0.20 with less than 30% sequence homology [38]. When five or more consecutive amino acids had torsion angles in the α-helical region of the Ramachandran plot, the hydrogen bonds typical of the α-helix were formed, and the resulting structure fit a very highly populated and finely focused area near ϕ = **-**63.8°, Ψ = **-**41.1°. No discussion of the implications of these non-classical torsional parameters on helical rise-per-residue or number of residues-per-turn appeared. A helix with ϕ = -62°, Ψ = -43° backbone torsion angles, bifurcated hydrogen bonds, and the helical parameters of the α-helix (**Fig 3**) is herein referred to as the experimental Némethy-, N- or 3.6_13/10_-helix. By examining the backbone torsional angles by nominal resolution, a compensatory shift in Φ, ψ was observed (**Fig 6**). This purely experimental perspective of protein helices determined at high resolution is consistent with bifurcated (three-centered) hydrogen bonds with a strong hydrogen bond to the *i+4* residue simultaneous with a weaker *i+3* hydrogen bond rather than the single linear hydrogen bond assumed by the classical helical models. What is surprising is that the observation of different average backbone torsional angles for protein helices was first reported by Blundell et al. in 1983 [34], and subsequently thereafter [38], without to our knowledge ever plotting the distribution of ϕ and Ψ (**Fig 5**).

**Table 1:** Hydrogen-bond analysis of the PDB as a function of nominal crystallographic resolution.

Resolution	Total H-Bonds	α-Helix Bonds	3_10_-Helix Bonds	β-Sheet Bonds	Shared 3-center H-bonds
0.00–0.49	101188	34594	14873	20531	14283
0.50–0.99	33446	9693	2345	12329	1980
1.00–1.49	718464	246901	57088	230275	47186
1.50–1.99	5342073	1942587	451007	1595126	375252
2.00–2.49	7739951	2936903	719304	2157150	626744
2.50–2.99	5178485	1922736	537574	1458120	505260
3.00–3.49	1839170	672369	202288	493697	210678
3.50–3.99	406006	141464	44390	109627	51326
4.00–4.49	137350	51589	15757	32724	18208
4.50–4.99	32332	12830	4077	6711	4205

### Averaged bimodal population vs. a single continuous population

In contrast to the usual expectation that the observation of non-classical backbone torsional angles was due to averaging of a bimodal distribution of many α-helices with a few 3_10_-helices, this study shows how the distribution of torsion angles in high-resolution torsion angles populated a single, continuous, smooth peak that is intermediate between the α- and 3_10_-helical forms. Whereas a bimodal population would be the expected result for helices that have some proportion of *i→i+4* hydrogen bonds (i.e., α-helical) and some proportion of *i→i+3* hydrogen bonds (i.e., 3_10_-helical), a single population is consistent with helices that are positioned to transition easily between *i→i+3* and *i→i+4* hydrogen bonds, and indeed have a significant portion with simultaneous three-center shared *i→i+3* and *i→i+4* hydrogen bonds. The evidence is consistent with a single consensus helical ensemble, the Némethy-, N- or 3.6_13/10_-helix, and a single local energy minimum, rather than two separate helical forms. This observation of non-classical helical torsion angles has appeared in the literature numerous times [38,40,41], but structural implications have apparently been largely ignored.

### Straight-forward explanations for persistence of an inaccurate helical models

The longevity and dominance of these two canonical helices in protein science is quite amazing, considering that two of Pauling’s key assumptions have been experimentally shown to be inaccurate. Bifurcated three-centered hydrogen bonds in proteins were found to occur with a frequency of 24% in 13 high-resolution (<1.6 Å) structures by Preissner et al. in 1991 [40]. McDonald and Thornton examined a dataset of 57 higher-resolution structures (< 2.0 Å) in 1994 and classified hydrogen bonding [42]. More significantly, the paper included diagrams and energetic estimates of three-centered hydrogen bonds by quantum mechanics with distributed multipole analyses. According to this analysis, four out of every five-backbone carbonyls failed to form three-centered hydrogen bonds, implying a frequency of 20% occurrence. This work was extended by Fain et al. in 2001/2008 on the presence of double and/or shared hydrogen bonds (estimated at approximately two-thirds of observed backbone hydrogen bonds and supported by quantum calculations) in the helices of globular proteins [43,44]. The discrepancy between the two studies probably arose from differences in the resolutions of the datasets examined and/or the choice of H-bonding parameters. Nevertheless, it is quite clear from these studies that amide three-centered hydrogen bonds are common features of high-resolution crystal structures of proteins and contradict the assumption of linear hydrogen bonds. Many have questioned the use of monopole electrostatics in molecular mechanics as misleading [20–22,45]. Use of multipole electrostatics and polarizability in X-ray refinement leads to significant improvements in the fits between model and electron density [28,29]. Cardamone et al. have recently published a critical review of multipole electrostatics, and their advantages over monopoles [46]. Nevertheless, protein crystal structures, from the originals by Kendrew [47] and Perutz [48] to recent additions to the PDB, have continued to utilized the two classic helices in their interpretation of electron density.

## Materials and Methods

### Hydrogen-bond statistics of the Protein Data Bank

Files were downloaded from the Protein Data Bank (PDB). A local PDB archive was created in 2008 and updated continuously through June 9, 2010, when this analysis was performed. A total of 60,755 proteins were examined, of which 53,040 were determined by X-ray crystallography (the main focus of this study); the remaining structures were determined by nuclear magnetic resonance (NMR) methods, electron microscopy, homology modeling, and other hybrid methods. Each structure was binned by nominal crystallographic resolution, cleaned, and pre-processed to fix structural anomalies and format inconsistencies. The 3D coordinates of main chain heavy atoms (i.e., ‘ATOM’ records) were not altered. Each of the resolution bins were then analyzed using programs written or adapted by Eric Welsh and Daniel J. Kuster as part of the StructureTools package [49], available from the authors under an open source (GPL) license. These scripts analyzed each crystallographic PDB file and generated a detailed list of hydrogen bonds. Secondary structures were then assigned based on the hydrogen-bonding patterns for each protein. Every helical hydrogen bond within each resolution bin was assigned to a category to give a total statistic for each resolution bin of the PDB as seen in Tables 1 and 2.

**Table 2:** Structural Percentage of the PDB as a function of nominal crystallographic resolution.

Resolution	α-Helix Bonds %	3_10-_Helix Bonds %	β- Sheet Bonds %	Shared Bonds %
0.00–0.49	34.19%	14.70%	20.29%	14.11%
0.50–0.99	28.98%	7.01%	36.86%	5.92%
1.00–1.49	34.37%	7.95%	32.05%	6.57%
1.50–1.99	36.36%	8.44%	29.86%	7.02%
2.00–2.49	37.94%	9.29%	27.87%	8.10%
2.50–2.99	37.13%	10.38%	28.16%	9.76%
3.00–3.49	36.56%	11.00%	26.84%	11.46%
3.50–3.99	34.84%	10.93%	27.00%	12.64%
4.00–4.49	37.56%	11.47%	23.83%	13.26%
4.50–4.99	39.68%	12.61%	20.76%	13.01%

### Hydrogen-bond detection

Hydrogens were added to each structure using the HBPLUS program of McDonald and Thornton [42], which was chosen after quantitative comparison of hydrogen bond geometries and visual inspection of several thousand hydrogen-bond placements for various algorithms. These criteria for hydrogen bonds would be relatively permissive for hydrogen bonds at large, but were well suited for the solvent-shielded interior of a protein helix. No extra constraints were placed on the detection of multiple hydrogen bonds—if atoms were close enough and within planar angles where hydrogen bond could occur, a hydrogen bond was detected. As a quality check, thousands of hydrogen-bond pairs were visually inspected using the SYBYL molecular modeling program; the severe steric constraints of helical backbone chain atoms made detection of geometric anomalies a straightforward (if tedious) task. Both random samples and a ranked list of the most extreme geometries were visually inspected to validate the procedure.

### Hydrogen-bond patterns define helices

Helices were assigned based on the pattern of i →i+3 and/or i →i+4 hydrogen bonds; a helix must have at least two consecutive helical hydrogen bonds (i.e., the shortest possible helical hydrogen bond pattern would be i →i+3 followed by i+1 → i+4). The final geometric parameters were sufficient to detect hydrogen bonds with zero anomalies/false positives. Tables 1 & 2 shows the statistics for each category by nominal X-ray resolution.

### Helical backbone statistics in the PDB appear to be converged

Repeated analyses of the helical hydrogen bond content of the PDB from 2008 to 2010 resulted in essentially identical plots [37]. The fact that neither the shape of distributions, nor the statistical moments of the distributions changed over this time suggested that the population of helical backbones in the PDB (more than 10 million observations of helical hydrogen bonds), was statistically converged, and unlikely to change either the details of analysis or conclusions discussed herein by analysis of more structures.

### Ramachandran plot of helical configurations by resolution

The classic two-dimensional Ramachandran-style plots of [φ,ψ] torsion angles were produced using a Matlab script. Millions of helical hydrogen-bond observations were clustered into 1° x 1° bins, allowing fine-grain frequency statistics to be calculated and visualized across all [φ, ψ] space. Distributions of ω torsion angles were also examined. As expected due to the planar nature of the amide bond, ω angles deviated very little from the 180° *trans*-conformation, so one can safely assume this invariant dimension does not contribute to helical backbone observations.

### Comparison with experimental neutron-diffraction data on proteins

Neutron diffraction allows the position of hydrogens to be determined more precisely. In contrast to X-ray diffraction experiments where the position of electron-sparse hydrogens are inferred from the coordinates of heavy atoms, neutron diffraction images hydrogen nuclei directly. The PDB had over 20 proteins structures determined by neutron diffraction. Improvements to hydrogen-bond orientation/geometry were seen when two DNA and a protein crystal structure were refined against joint neutron/X-ray diffraction data sets using a force field derived with AMOEBA multipole electrostatics [50].

Different proteins (PDB Codes = 1L2K, 1L2N, 1NTP, 1VCX, 1WQ2, 2DXM, 2GVE, 2INQ, 2MB5, 2R24, 2VS2, 2WYX, 2YZ4, 2ZOI, 2ZWB, 2ZYE, 3A1R, 3BYC, 3HGN, 3INS, 3KKX, 3KMF, 3KYX, 3L45, 5PTI and 5RSA) whose structures had been determined by neutron diffraction were analyzed for their hydrogen-bonding patterns. This set contained 3659 backbone H-bonds in the 26 proteins. Structure Tools [49] was used to detect and analyze hydrogen-bond content according to geometric rules. As can be seen by the statistics in Table 3, shared H-bonds account for 18.5% of the overall total of 1711 helical H-bonds detected. Thus, although the studies by neutron diffraction studies are limited compared to X-ray crystallography, the ability of neutron diffraction to directly locate the amide hydrogen strongly supported a significant role for shared H-bonds in protein helices.

**Table 3:** Hydrogen-bond analyses for neutron-diffraction data for 26 proteins.

Total H-Bond Summary	Total H-Bond Summary By Percentage
Total i to i+4 bonds: 1329	Total i to i+4 bonds: 36.3%
Total i to i+3 bonds: 382	Total i to i+3 bonds: 10.4%
Total H-Bonds in Helices = 1711	
Total β-Sheet bonds: 1043	Total β -Sheet bonds: 28.5%
Total # of bonds at bends: 431	Total # of bonds at bends: 11.8%
Total # of bonds at turns: 26	Total # of bonds at turns: 0.7%
Total # of bonds at coils: 302	Total # of bonds at coils: 8.3%
Total Disallowed bonds: 146	Total Disallowed bonds: 4.0%
Summary—3659 H-bonds detected = 100%	
Bifurcated H-bonds: 317	% Bifurcated H-Bonds in Helices = 317/1711 = 18.5%

### Methods for Calculating Helical Parameters

**Fig 7:**
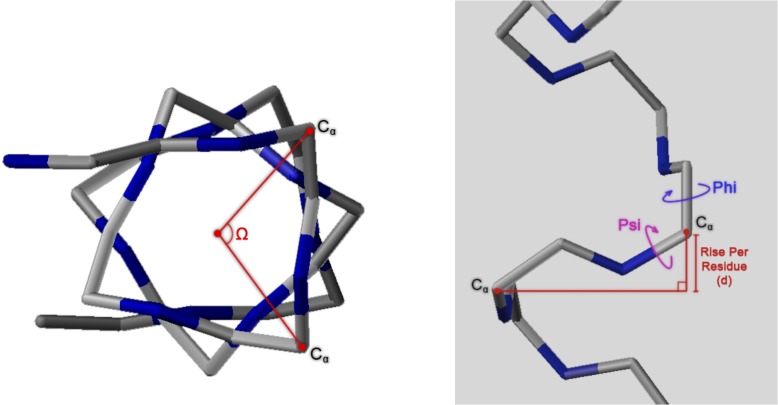
Definition of helical parameters with an oligopeptide helix backbone (nitrogens in blue). Left: Helix winding (Ω) is the angle between two adjacent Cα vectors (**n** = residues-per-turn/360). Right: Helix pitch (**p**) is the rise-per-residue, or vertical distance between Ω adjacent residues, projected on the helical axis.

**Fig 8:**
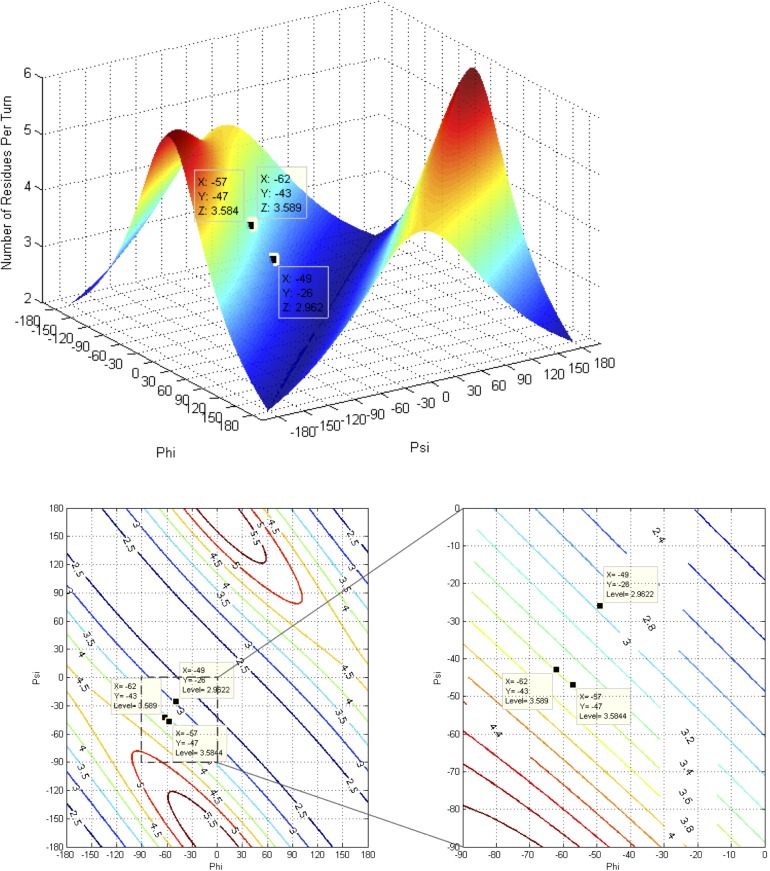
Contours and 3D surface helical pitch (n d) overlaid on 2D φ, ψ plot. Positions of classic α-helix (φ = −57, ψ = −47), 3_10_-helix (φ = −49, ψ = −26), and experimental Némethy-, N- or 3.6_13/10_-helix (φ = −62, ψ = −43) are indicated. Note that the helical pitch is nearly identical for the α- and 3.6_13/10_-helices—they lie on a contour of essentially equal value.

**Fig 9:**
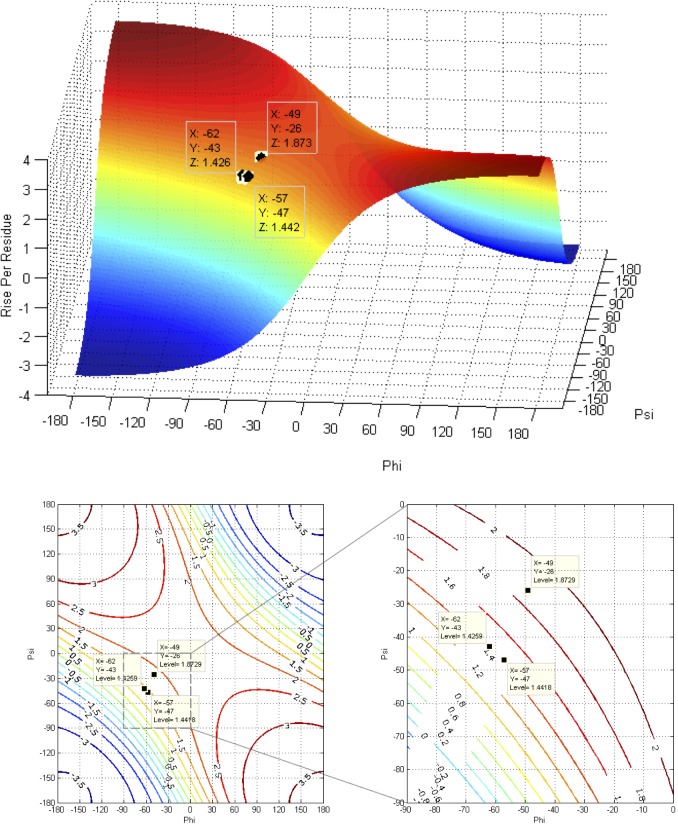
Contours and 3D surface showing rise-per-residue (d in Å) overlaid on 2D φ,ψ plot. Positions of classic α-helix (φ = −57, ψ = −47), 3_10_-helix (φ = −49, ψ = −26), and Némethy-, N- or 3.6_13/10_-helix (φ = −62, ψ = −43) are indicated. Note that **d** is approximately the same for the α- and 3.6_13/10_-helices.

**Fig 10:**
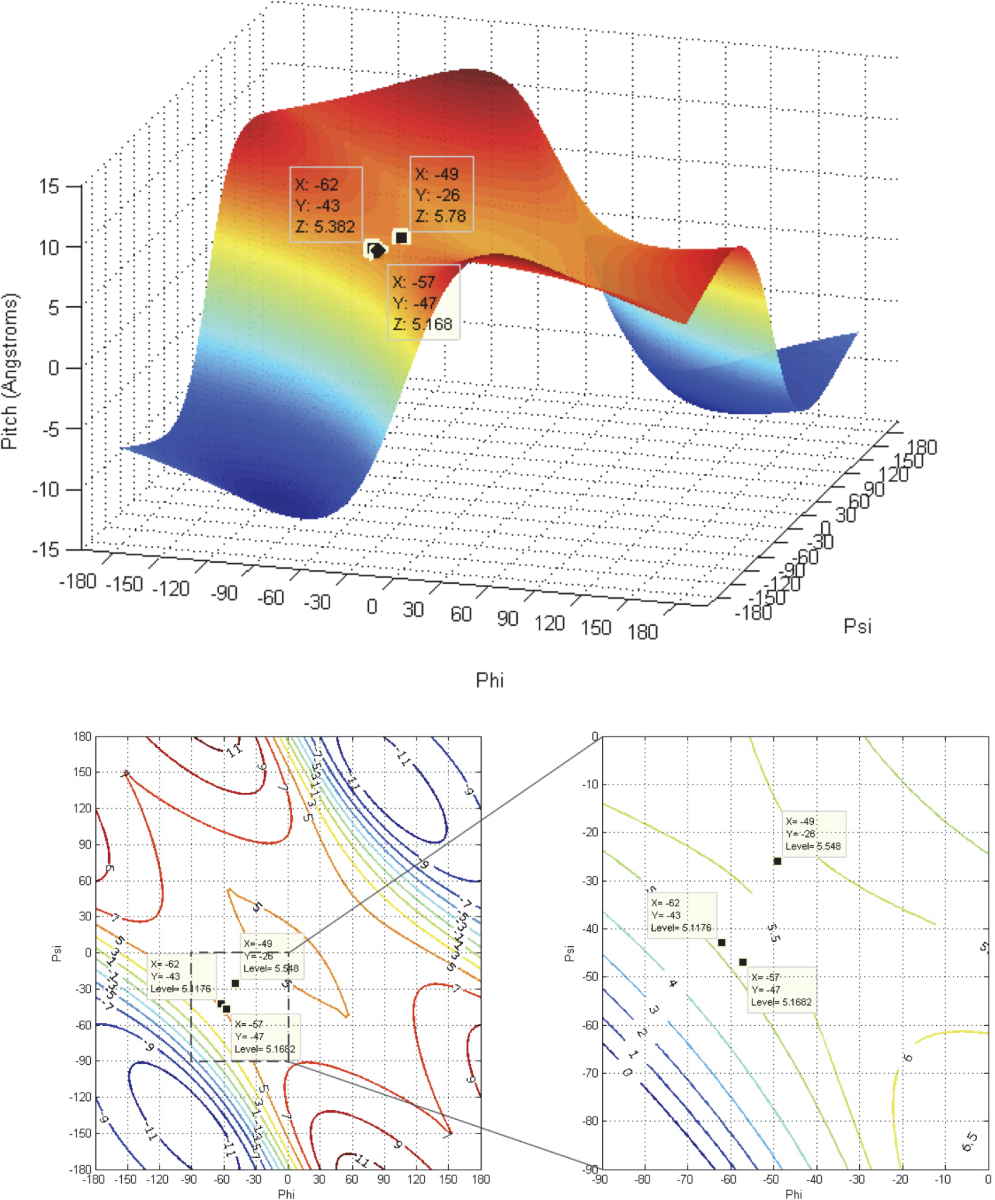
Contours and 3D surface showing number of residues-per-turn (n) overlaid on 2D φ, ψ plot. Positions of classic α-helix (φ = −57, ψ = −47), 3_10_-helix (φ = −49, ψ = −26), and experimental Némethy-, N- or 3.6_13/10_-helix (φ = −62, ψ = −43) are indicated. Note that **n** is approximately the same for the α- and 3.6_13/10_-helices.

The mathematics used to analyze the impact of changes in backbone torsion angles on helix pitch and orientation of Cα-Cβ vectors in proteins was described by Ramachandran et al. [51]. A more recent and thorough generalization has been made based on quaternions [52]. The following equations were used to analyze the impact of the backbone torsion parameters Φ, Ψ on helix pitch (**p**) and residues per turn (**n**) (**Figs 7, 8, 9 and 10**):

Model equations used for helix-winding relationships:

Helical pitch (**p**, rise per turn) = **d** (Rise per residue) * **n** (Residues per Turn)

Relationships exists between Φ,ψ and **Ω** for calculating **n** and **d**. ^*1*^

$$\begin{array}{r} s=\frac{\Phi +\Psi }{2},t=\frac{\Phi -\Psi }{2}\\ \cos\left(\frac{\theta }{2}\right)=\;-0.817\sin\left(s\right)+0.045\;\text{sin}\left(t\right) \end{array}$$ 2

θ can then be used to calculate **n** and **d**:

$$\begin{array}{r} n=360/\theta \\ d\;sin\left(\frac{\theta }{2}\right)=\;-0.68\cos\left(t\right)+2.9\;\text{cos}\left(s\right) \end{array}$$ 3

- θ —Rotation Angle per Residue in Trans
- ϕ —Phi, Torsion Angle of N-C_α_
- ψ —Psi, Torsion Angle of C_α_-C'

Thus, helical pitch (**p**) can be expressed directly in terms of Ψ and Φ.

p = d x n

- ^*1*^_*θ*, *Ψ*, *and Φ are expressed in radians*._
- ^*2*^_*resulting pitch is expressed in Angstroms per turn*._

### Force-field dependence

Because peptides are so inherently flexible and multiple helical forms have been suggested depending on the experimental/theoretical approach, the choice of force field for the MD simulations was crucial. In particular, force fields dependent on monopole electrostatics only reinforce Pauling’s assumption of linear hydrogen bonds. A previous study by Zheng et al. [30] compared three monopole force fields in common use with AMOEBA, a second-generation force field using multipole electrostatics and polarizability [26]; a significant improvement in the ability of the AMOEBA force field to predict experimental observations on peptide dynamics was observed and justified the choice of AMOEBA for the MD simulations.

### Isothermal-isobaric MD simulations

All solvent-solute systems were optimized via the TINKER MINIMIZE program with an 0.01 kcal/mol/Ang RMS gradient cutoff prior to dynamics simulation. All MD simulations were performed using either the Ponder lab compute cluster or compute nodes at the Washington University Medical School Center for High Performance Computing. Since the replica-exchange MD simulation only offered structure samplings, rather than true dynamic trajectories, a total of 8 isothermal-isobaric simulations for oligoalanine helices at 300° K and under 1.0 atmospheres were run for 17 ns to obtain trajectory data. The vdw cutoff was set to be 12.0 Å and the particle-mesh Ewald real-space cutoff to 7.0 Å. Induced dipoles were converged to an RMS change of less than 0.01 Debye per iteration. The simple bonded/non-bonded RESPA integration scheme [53] was used to enable a larger simulation step size of 2.0 fs. Trajectory frames were recorded every 1 ps. The resulting hydrogen-bond matrix was compared to the matrix from the replica-exchange, and found to be reasonably similar suggesting that trajectory sampling was adequate.

### Replica-exchange MD

Replica-exchange MD simulation was performed with the protocol of Sugita and Okamoto [54] to get extensive structural sampling for the oligoalanine helices. Twenty-four replicas exponentially distributed from 300° K to 400° K were established with the help of an automated temperature generator for REMD-simulations [55] to obtain an exchange rate near 20% for each structure. The simulation was carried out with the TIREX replica-exchange program developed by Penev et al. [56]. All the setups were the same as the isobaric-isothermal simulations as described above, except the NVT ensemble was used and the trajectories were recorded every 0.5 ps. Each replica was simulated for 10 ns with exchanges attempted every 1000 steps.

### Molecular dynamics simulations of crambin in water [37]

The protein crambin from PDB entry 1CRN [57] was hydrated in a periodic box of explicit water. For the AMOEBA simulation, the AMOEBA water model [18] was employed. For CHARMM and OPLS-AA simulations, the TIP3P water model [58] was used. Simulation data was collected over tens of nanoseconds and the resulting structures analyzed for changes in coordinates.

### AMOEBA molecular dynamics simulation of oligoalanine in water

The starting alanine helix of 12 residues was directly built using the TINKER PROTEIN program with Φ = -60° and Ψ = -43° torsion angles to generate a right-handed α-helix with an N-terminal ACE (acetyl) and a C-terminal NME (N-methyamide) capping groups. After minimization by TINKER MINIMIZE with the AMOEBA force field with a 0.01 cutoff, the final torsional angles were approximately (-63°, -39°) similar to ones observed in high-resolution crystal structures. The TINKER XYZEDIT program was used to tailor a water box to solvate the starting helix. Since the TIREX replica exchange program offered only NVT options, the box boundaries were manually adjusted to achieve an average pressure close to 1 atm at 300° K resulting in a water box with 40.7777 Å on each side.

### AMOEBA molecular dynamics simulation of oligo-α-aminoisobutyric acid (Aib) in water

The AMOEBA force-field parameters for Aib were constructed with the described protocol [27] by fitting to the Gaussian09 quantum calculation of an Ac-Aib-NHMe dipeptide. A right-handed, 12-residue, α-helical capped oligo-α-aminoisobutyric acid was built the same way as the oligoalanine helix. The water box was set to be 40.787 Å on each side to achieve a pressure close to 1 atm. The replica exchange, trajectory extraction, and Ramachandran map were done the same as for oligo-alanine mentioned above.

### Energy surface sampling in the α-helical region

Ace-Ala-Ala-Ala-NMe structures in the range of φ = [-100 to -50] and ψ = [-50 to 0] were constructed with TINKER PROTEIN package at 1 degree increments for force field and 2 degree increments for quantum mechanics calculations. Then, their energy was computed with AMOEBA09, OPLS-AA, OPLS-AAL, CHARMM22, AMBER99sb, and with the MP2/6-311(1d,1p) quantum basis set. The computations were done on the tripeptide *in vacuo* with the permutations of φ, ψ angles applied to all three Ala residues simultaneously.

### Analysis of protein helices by nominal crystallographic resolution

Analysis of all experimental structural models of helices in proteins in the Protein DataBank (Tables 1 and 2) exposed subtle inconsistencies in the torsional angle parameters of experimental high-resolution models compared to the classic α− and 3_10_-helical models [37]. At high resolutions, where the experimental electron density accurately constrained helical conformations with the need for conformational assumptions and/or force field constraints, the backbone torsional angles populated a smooth distribution of helical conformations with a single maximum at φ = -62, φ = -43 (**Fig 4**). This is in contrast to the anticipated bimodal distribution that would result from a mixture of classic α− and 3_10_−helices. Hovmoller et al. previously analyzed a non-redundant set of 1042 protein subunits from the PDB of Jan-2002; each structure was determined by X-ray diffraction to a resolution of 2.0 Å or better, refined to R ≤ 0.20, and had less than 30% sequence homology [38].

The historical use of monopole electrostatics in force fields used to build atomic models from lower-resolution electron density appears (**Fig 5**) to have biased the PDB with early protein structures in which the presence of linear hydrogen bonding is over-represented. While the magnitude of changes in φ,ψ backbone torsion angles required to accurately fit high-resolution crystallographic data is small (i.e., only a few degrees), these errors can have a dramatic impact on other aspects of atomic structural models, from helical parameters propagating error at each helical turn, to significant errors in dipole moments, and most critically, upon the thermodynamics of interaction with helical surfaces. In order to address these issues, the geometrical relationships (**Eqs 2 & 3**) between backbone torsion angles and helical parameters that impact Cα − Cβ vectors were revisited.

### Impact of helical parameters

The almost unanimous support for the two classical helices by protein crystallographers over several decades was a serious concern. If backbone angles were really different and a strongly coupled relationship existed between helical parameters and backbone torsional angles, then changes in helical parameters should have been detected many times; if not in early crystal structures, then surely in the many tens of thousands of models that have been validated since! Starting with fundamental definitions for helical conformations, the effect of observed changes as a function of backbone torsional angles were plotted in terms of number-of-residues-per-turn (**n**) and rise-per-residue (**p**) (Fig 6). The implications of Figs 7, 8, 9 and 10 are profound, and may help explain why the classical α-helix has enjoyed ongoing support in the crystallographic community.

The helical parameters (**n,p**) for the experimental Némethy-, N- or 3.6_13/10_-helix are identical to those of the classical α-helix, and unless the crystallographic resolution for a particular experiment is exceptional (i.e., < 1.5 Å), then the electron density associated with the carbonyl oxygen of the amide was insufficient to discriminate between helical structures. The use of inaccurate electrostatic treatments (i.e., monopole force fields), has also been a confounding issue, due to historical refinement methods that employing these potentials. In other words, the electron density from the heavy atoms of the peptide backbone (i.e., carbonyl carbon, amide nitrogen, Cα, and Cβ) occupied identical positions in space for the classical α-helix and the experimental Némethy-, N- or 3.6_13/10_-helix. Only the highest-resolution structures would be expected to discriminate between the two based only upon the position of the carbonyl oxygen, and such differences would be subtle. Initially, observations of the high-resolution helical population were assumed to impact the overall helical parameters, which seemed reasonable as the high-resolution population is intermediate between the α- and 3_10_-helical populations and the 3_10_-helix is longer and tighter than the α-helix. However, theoretical analysis of the dependence of helical parameters on torsional angles (**Figs 7, 8, 9, and 10**) resolved this apparent dilemma.

A compensatory relationship between φ and ψ due to a crankshaft-like motion [39] of the planar amide bond between the two torsions allows orientation of the carbonyl oxygen semi-independently from the height, pitch, and width of the helix. To accommodate the rotation of the amide bond about an axis connecting the two α-carbons (crank-shaft rotation), minor adjustments to bond angles are required, but energetic constraints on bond angles are comparatively low. This rationalizes the observations of three-centered helical hydrogen bonds in high-resolution protein structures that have been previously annotated/modeled as α-helices [9].

### Comparison with protein helices from high-resolution crystal structures

**Fig 11:**
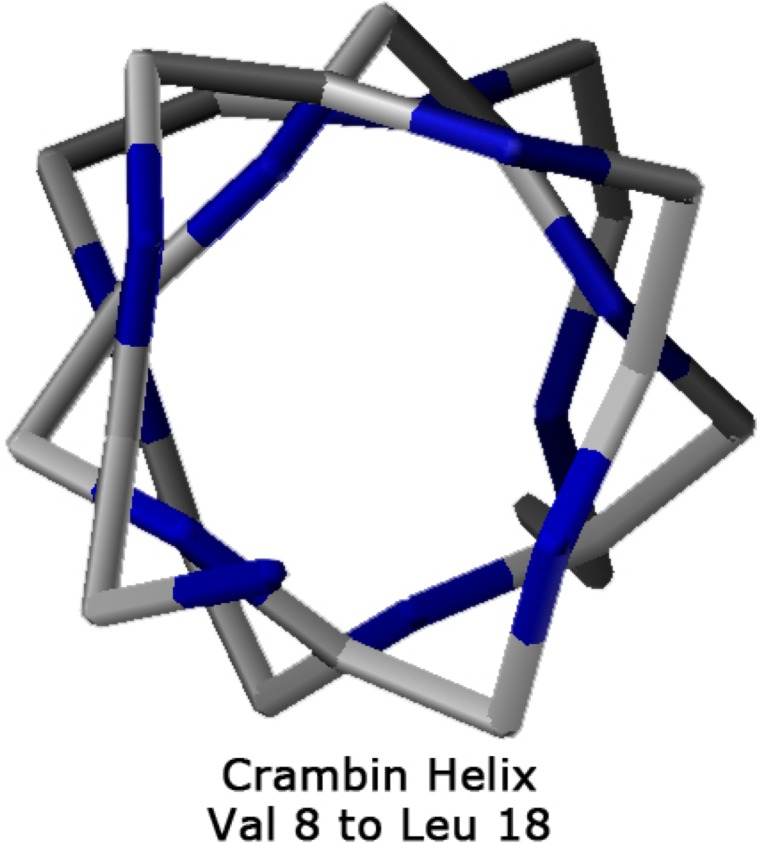
Helix 8–18 from the crambin crystal structure [PDB:1ejg], viewed from above the N-terminus. The average φ value along the helix was between the experimental Némethy-, N- or 3.6_13/10_-helix and the α-helix. However, the average ψ value was between the experimental 3.6_13/10_-helix and a 3_10_-helix.

Protein helices from experimental data with the most precise X-ray determination of atomic positions supports the analysis of Némethy-, N- or 3.6_13/10_-helices. The impact of torsion parameters on helix geometry can be independently accessed because the abundance of observations ensures that a very high-resolution structural model is built from experimental data with minimal contribution from geometric/energetic constraints. The small protein crambin with 46-residues has helices from experimental data obtained at very high resolution (PDB = 1ejg, O.54 Å)[59]. A more highly refined crambin structure (PDB = 3nir, O.48 Å) has recently been reported [60]. In Table 4 are listed the experimental Φ,Ψ torsion values for helix (7–18) of crambin 1ejg (**Fig 11**). While the helical structure is obvious, the backbone torsion angles of the helical residues show considerable variation implying that they are sensitive to their environment, once again supporting the analysis of Hollingsworth et al. that secondary structures in proteins are not linear groups [32]; i.e., protein secondary structures do not behave as homopolymers with identical conformational parameters per residue. The shared hydrogen bridges in helices facilitate environmental adjustment by reducing the energetic barriers to optimize backbone torsional angles for side-chain interactions, and to respond to the local electrostatic environment [61]. In fact, MD simulations indicate that even short homopolymers exhibit dynamics heterogeneity in conformational space [62].

**Table 4:** Backbone Torsional Angles of High-resolution (1ejg, 0.54 Å) Structure of Crambin 7–18 Helix (Fig 11).

Residue #	Amino Acid	Φ	Ψ	Φ Deviation	Ψ Deviation
7	Ile	-52.6	-53.6	7.9	-11.7
8	Val	-53.2	-49.3	7.3	-7.4
9	Ala	-61.1	-42.0	-0.6	-0.0
10	Arg	-63.0	-44.6	-2.6	-2.7
11	Ser	-60.1	-42.1	0.4	-0.2
12	Asn	-60.7	-43.8	-0.3	-1.9
13	Phe	-59.7	-45.6	0.8	-3.7
	Average	-60.5	-41.9		
	N-Helix	-62	-43		

### Analysis of molecular dynamics simulations

**Fig 12:**
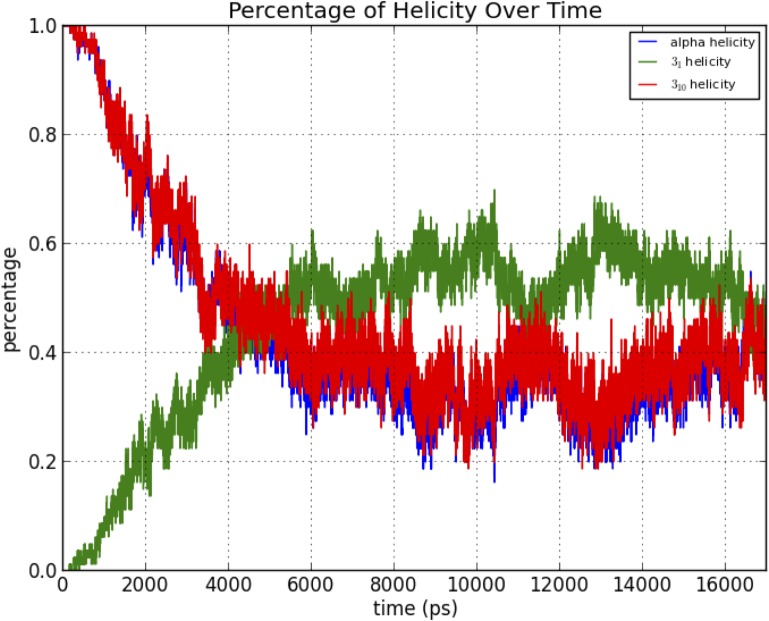
Transition of oligoalanine from its starting α-helical conformation to intermediate states that includes near α-, 3_10_- and polyglycine conformations.

**Fig 13:**
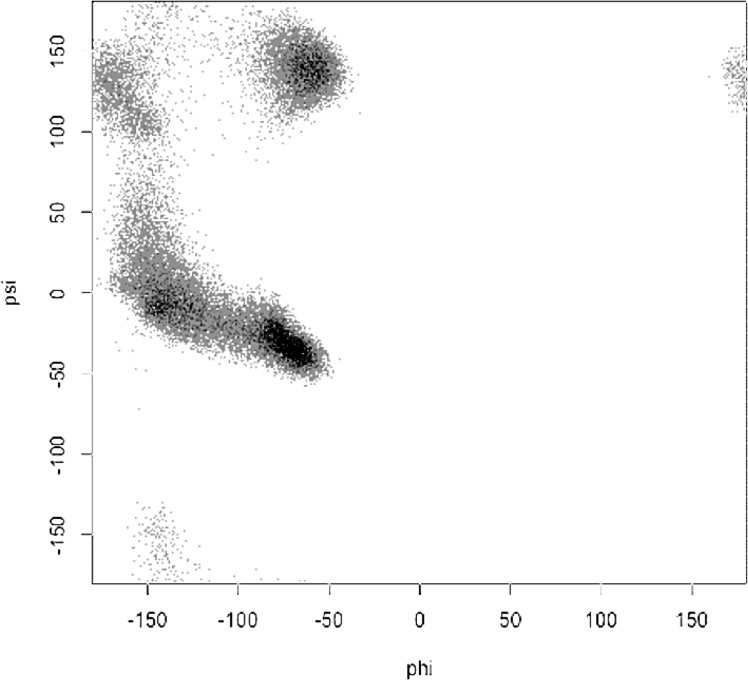
Ramachandran plot of backbone torsional angles from the AMOEBA BIO09 MD simulation of 12-residue oligoalanine.

**Fig 14:**
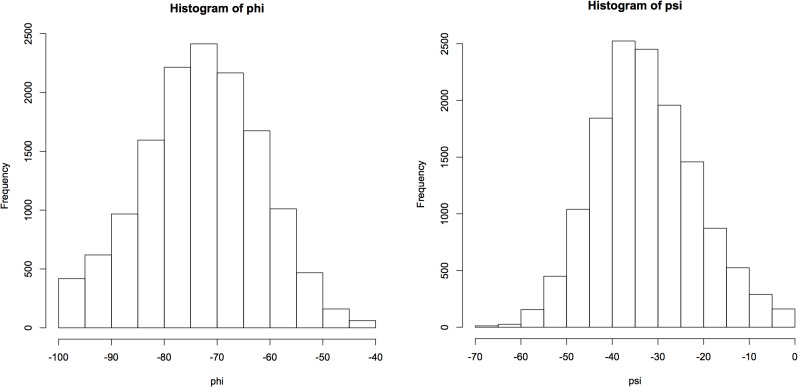
The Φ, Ψ angles distribution of the data filtered with H-bond distance (<4 Å) and Φ, Ψ angles range (-100 -> 0, -70 -> 0). The median of Φ, Ψ angles was Φ-72.8, Ψ-33.4.

**Fig 15:**
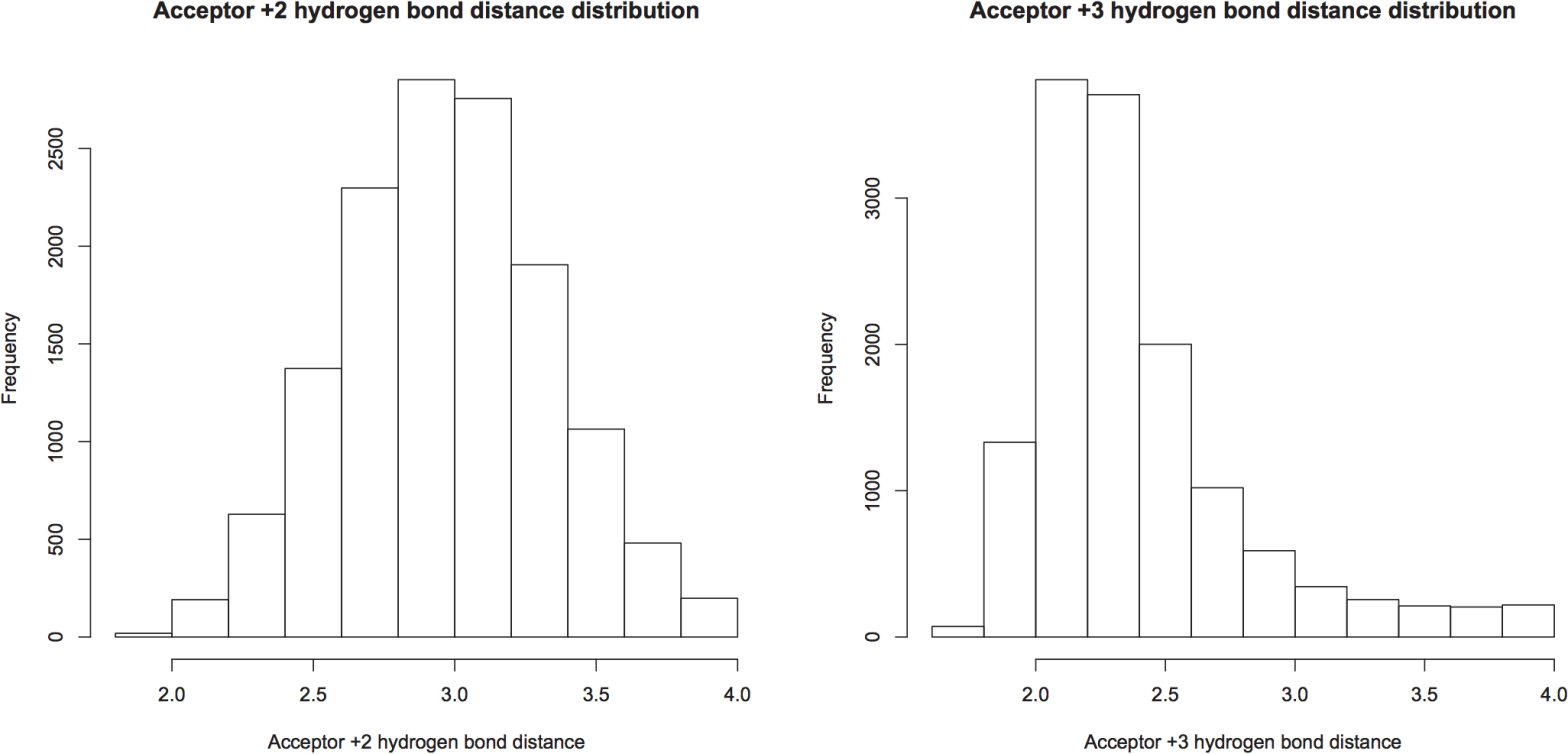
Distance distribution of observed H-bonds filtered by H-bond distance (<4 Å) and Φ, Ψ angles range (-100 -> 0, -70 -> 0). The medians of *i+3* and *i+4* distances were 2.97 Å and 2.28 Å, respectively.

**Fig 16:**
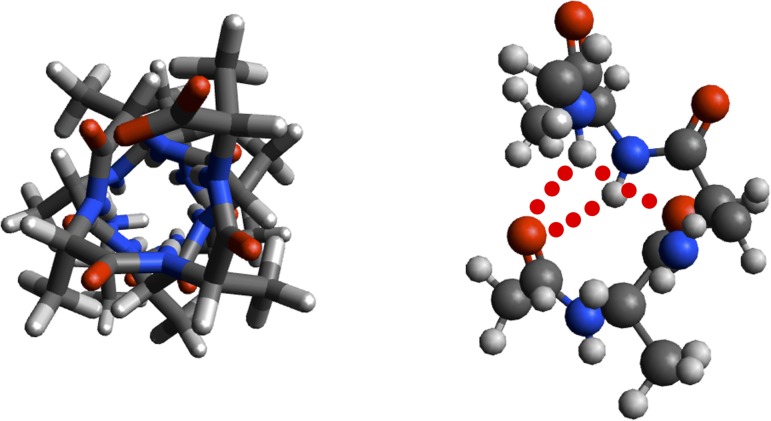
Orthogonal views of ball-and-stick model of “dynamic” helix of 12-residue capped oligoalanine with two three-centered hydrogen bonds indicated (red dots).

As the structure was started in a helical conformation, this was confirmed by monitoring the changes in conformation as a function of length of simulation (**Fig 12**). A Ramachandran plot from the MD simulation demonstrated significant sampling of torsional space different from the starting helical conformation to provide evidence for sufficient sampling (**Fig 13**). Only a single peak was observed in the region of φ = [-100 → 0], ψ = [-70 → 0] region. To further determine the center of the structures, structural filtering based on α- and 3_10_-helical hydrogen bond lengths (i.e., < 4 Å) and φ,ψ angles was performed (**Fig 14**). Both φ,ψ angles and *i→i+4* distances followed normal distribution with slight skewing to the left of *i→i+4* hydrogen bonds. Considering the strong penalty for steric overlap, an approximately normal distribution resulted. The “average” structure had helical backbone torsions φ = −72.78, ψ = −33.43 and *i→i+3* = 2.97 Å; *i→i+4* = 2.28 Å hydrogen-bond distances (**Fig 15**) with a conformation that resembled the α- and experimental Némethy-, N- or 3.6_13/10_-helix (**Fig 16**).

More detailed analyses of hydrogen-bonding dynamics from an oligoalanine MD simulation with AMOEBA can be found in a paper by Liu et al. [62] comparing the relative stabilities of helices comprised of capped 12-residues peptides of oligoglycine, oligoalanine and oligo-β-alanine.

### Quantum calculations on Ac-Ala-Ala-Ala-Nme

In order to observe any significant electronic differences between α-, experimental Némethy-, N- or 3.6_13/10_- and this dynamic-helix, MP2/6-311(1d,1p) quantum calculations were performed on a simple capped tripeptide model, Ac-Ala-Ala-Ala-NMe, first *in vacuo*, and then with the PCM hydration model [63]. The relative energies and minima locations of the three helical conformers are given in Tables 5 & 6. The electron densities of the experimental Némethy-, N- or 3.6_13/10_- and dynamic-helices were very similar. But in the classic α-helix, HN39 was slightly more polarized with its electron density oriented towards O3.

**Table 5:** Relative energies of the three helical structures of Ac-Ala-Ala-Ala-NHMe by MP2/6–311(1d,1p) quantum calculations *in vacuo*.

	Energy/Eh	Relative Energy/ kcal
classic α-helix	-988.0102	1.958
Némethy-, N- or 3.6_13/10_-helix	-988.0134	0.00
“dynamic”	-988.0126	0.501

**Table 6:** Location of the two energy mimima-like positions on the potential energy surfaces of Ac-Ala-Ala-Ala-NHMe based on methodology.

	Position of the minimum	Position of the second funnel
MP2/6-311(1d,1p)	Φ = −70, Ψ = −17	Φ = −73, Ψ = −33
Amber99sb	Φ = −69, Ψ = −16	Φ = −71, Ψ = −34
AMOEBA BIO09	Φ = −69, Ψ = −37	N/A
CHARMM22	Φ = −75, Ψ = −30	Φ = −78, Ψ = −20
OPLS-AA	Φ = −74, Ψ = −33	Φ = −71, Ψ = −16
OPLS-AAL	Φ = −75, Ψ = −30	Φ = −79, Ψ = −11

A large decrease in energy for the α-helical region was observed by the PCM calculation, which lowered its minimum to 0.45 kcal/mol lower than the 3_10_-helix (approximately a 2:1 ratio at room temperature) with an energy barrier of 0.7 kcal/mol (~kT) between the 3_10_ and α-helical regions. This can be explained by the interaction energy of PCM water with the hydrophilic groups on the capped tripeptide that were not hydrogen bonded, and thus a compensation of the strong *in vacuo* end effects. But besides this effect, the center of the 3_10_-helical well also shifted from (-70, -15) to (-65, -20), a more depolarized, shorter *i→i+3* distance and similar *i→i+4* distance structure. The α-helical well was now centered about (-65, -37). Both centers shifted to tighter and more polarized structures than *in vacuo*. It is well-known that stability of α- versus 3_10_-helices in short peptides depends strongly on length and dielectric of the solvent [64]. This arises primarily from the extra hydrogen bond in a classical 3_10-_helix versus an α-helix of the same length. Further QM calculations on peptides of different lengths are required to resolve the end effects seen with the tripeptide.

### Estimated dipole moments for different helical conformations

To compensate for the strong end effects of the short tripeptide model, capped twelve-residue oligoalanines were generated with backbone torsion angles set to those of α-helix, 3_10_-helix, experimental Némethy-, N- or 3.6_13/10_-helix, the MD helix and the QM helix. PDB files of these structures were submitted to a web-based server for Adaptive Poisson-Boltzman Solver described by Felder et al. [65] to calculate protein dipoles and the results are presented in Table 7.

**Table 7:** Calculated dipole moments [65] in Debye using a modified set of parameters for solvation energy (Parse) partial atomic charges [67] for various capped 12-residue oligoalanine helices as well as for crambin and its isolated helix compared with those using AMOEBA multipoles.

α-helix	74 vs. 60.2	3_10_-helix	70 vs. 62.1
Némethy-, N- or 3.6_13/10_-helix	70 vs. 62.1	MD-helix	72 vs. 55.4
A-Helix	72 vs. 55.6	QM-helix	67 vs. 51.9
Crambin	54 vs. 64.6	Crambin helix (Ac-6-19-NHMe)	88 vs. 70.9

The experimental 3.6_13/10_-helix has a calculated dipole moment most similar to the 3_10_-helix reflecting the reorientation of the amide bond from that of the classic α-helix. Sengupta et al. reported a calculated dipole moment of 42.7 Debye for a oligoalanine α-helix of twelve residues using CHARMM monopole charges [66]. For comparison, TINKER was used to calculate the dipole moments using AMOEBA multipole charges. The differences in magnitude (**Table 7**) are significant, and the relative values change as well with a maximum difference of 7 Debye (74–67) for the dipoles from the server, and 10.2 Debye (62.1–51.9) for AMOEBA dipoles. Obviously, the redistribution of charge using multipoles versus monopoles significantly impacts the estimation of dipole moments. In order to further verify significant differences between the approaches, dipole moments were calculated for crambin (PDB: 3nir, O.48 Å) and its isolated helix (Ac−6−19−NHMe). The switch in relative magnitudes between dipole moments calculated with a monopole approximation versus a multipole method for crambin and its isolated helix (**Table 7**) dramatically emphasize the importance of multipoles in electrostatic interactions.

Ripoll et al. analyzing the alignment of peptide-bond dipole moments with the local electrostatic field generated by the rest of the molecule for a large set of native proteins; dipole moments associated with α-helical conformations show the best alignment with the electrostatic field [68]. The large difference in dipole moment seen for the different helical conformations suggests that the electrostatic environmental adjacent to a protein helix could modulate its backbone torsional angles with minimal perturbation to side-chain orientations and their steric interactions.

### *In vacuo* comparisons of helical minima and activation energies for transitions

**Fig 17:**
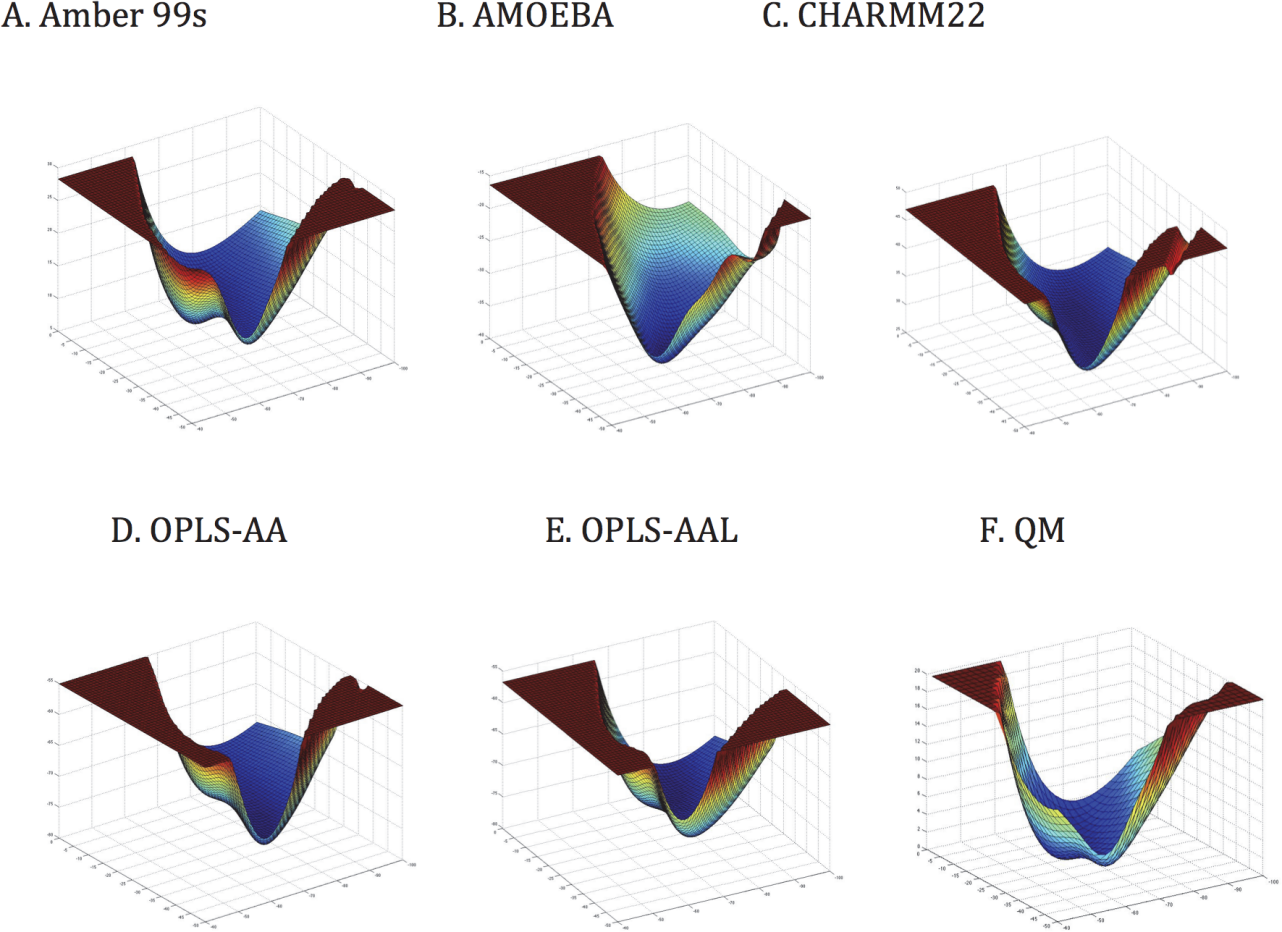
Potential surfaces for torsional force-fields surfaces surrounding helical conformations for A. AMBER99sb, B. AMOEBABIO09, C. CHARMM22, D. OPLS-AA, and E. OPLS-AAL versus F. QM—MP2/6-311(1d,1p) basis set.

As a baseline for comparison, the potential surfaces around helical conformations of Ac-Ala-Ala-Ala-NMe were generated *in vacuo* using the three monopole force fields and AMOEBA. These were compared with MP2/6-311(1d,1p) quantum calculations that sampled the same grid points (ϕ = -100 → 0, ψ = -70 → 0). Energies greater than the minimum by more than 20 kcal/mol were normalized to make the picture clearer. The six plots (**Fig 17**) show potential surfaces around the helical conformers, which clearly demonstrate significant differences in conformer stability and the limited activation energy barriers between them.

**Fig 18:**
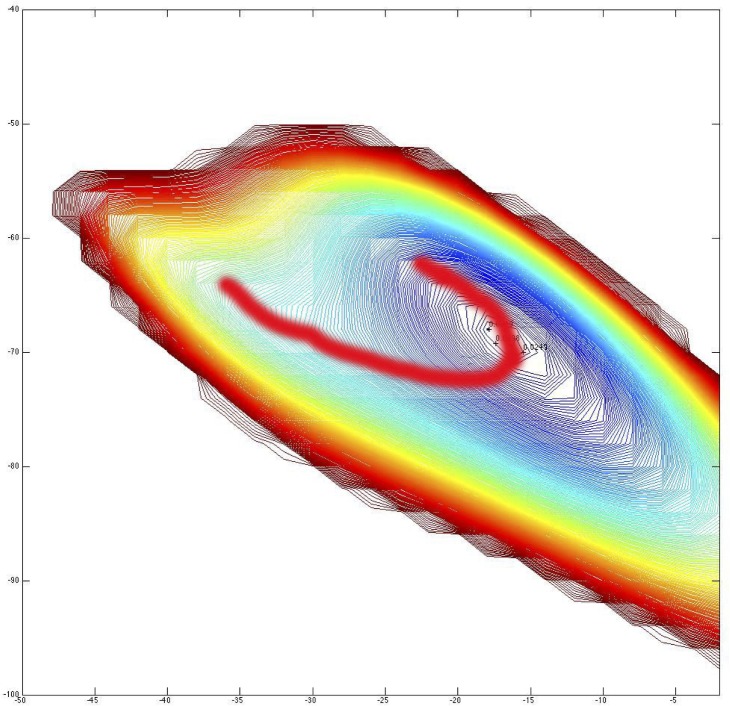
The potential surface near helical torsional angles for Ac-Ala-Ala-Ala-NMe as calculated by QM. The red line traces the transition between the two energy minima-like conformers without an activation energy barrier.

To further characterize these potential surfaces, the locations of the activation energy required for transitions between minima were tabulated. On the contour plots, both QM and the monopole force fields show potential funnels with two minima-like positions. From the contour plots for all the potential surfaces, no energy barrier existed between the two minima, and a “U” shape path would lead the structure from the higher energy position to the lower energy one without any activation-energy barrier (**Fig 18**). By QM calculations, the minimum energy-like conformer by QM was located at ϕ = -70, ψ = -17, while the other conformer was found at φ = -73, ψ = -33. The first was close to the classic 3_10_-helix and the second closer to the “dynamic” helix. Thus, they approximate the 3_10_ and α-helix regions, respectively. This suggests that the 3_10_-helix was energetically the most stable structure for Ac-Ala-Ala-Ala-NHMe *in vacuo*. This is plausible since the more compact hydrogen-bonding pattern of the 3_10_-helix allows one more hydrogen bond than the α-helix for the same length of peptide [64].

The QM potential well (**Fig 18**) supports the absence of two canonical helices. Whereas there appear to be two minima on the QM plot, the activation energy barrier between them, if any, was so low that they are functionally merged. All of the calculated potential surfaces (**Fig 17**) have transitions between the two detected minima similar to that of the QM surface. Thus, none of the potentials required a peptide to overcome an activation barrier between helical minima supporting the conclusion that there is no basis for the classical α- and 3_10_-helix dichotomy, and supporting the hypothesis of the “molten helix” suggested in 1995 by Smythe et al. [69,70]. In addition, the AMOEBA surface (**Fig 17(B)**) most resembles the QM surface (**Fig 17(F)**) in depth of the potential well, but differs slightly in absence of distinct minima corresponding to the two helical conformations. Despite direct parameterization using QM, the AMOEBA potential surface does not exactly duplicate that from QM suggesting that further improvements in parameterization can still be made.

### Evidence from high-resolution crystal structures of peptides supporting α- and 3_10_-helices

Abundant high-resolution crystal structures of small peptides clearly show the two classical helices [71–73] as well as transitions between them depending on sequence [74,75] and crystallization conditions [76,77]. In most of these cases, the crystalline peptides contained one, or more, *gem*-dimethyl amino acids, such as α-aminoisobutyric acid (AIB, α-methylalanine) or another amino acid in which the α-proton was replaced by a methyl group. Aib is highly helicogenic and surveys of synthetic Aib-containing peptides show a preponderance of 3_10_-, -, and mixed 3_10_/α-helical structures. Aib residues have been observed in non-helical conformations, primarily into the polyproline II (P(II)) and fully extended regions of conformational space [78]. The achiral Aib residue can adopt both left (α(L))- and right (α(R))-handed helical conformations. These experimental observations confirm the theoretical predictions of Marshall and Bosshard on the impact of methyl for proton substitution on peptide backbones made in 1972 [73].

The resolution obtained by direct methods on small molecules exceeds that normally obtained for proteins. A strong argument can be made that the data from Aib-containing helical peptides overrules the rejection of the two classical helices based on data from the PDB. To quote from an early study of Aib oligomers by Paterson et al. [79], “Tetrahedral symmetrical geometry for these substituents favors the α-helical conformation for the α-aminoisobutyric acid residue (and di- and tripeptides thereof) whereas asymmetric geometry, derived from well-refined X-ray structures, gives the 3_10_ conformation as the preferred structure. Analysis of pairwise atomic interactions indicates that favorable backbone-backbone interactions lower the overall energy of the molecule in the 3_10_ conformation when the substitution on the C^α^ atom is asymmetric.”

There is, however, a plausible explanation of the observations seen with Aib-containing helical peptides; the presence of the *gem*-dimethy groups on the peptide backbone favor the 3_10_-helix due to steric interactions [80]. In fact, Hodgkin et al. proposed a 3.6_10_-helix in 1990 [80] based on the structure and hydrogen-bonding pattern of Tos-(Aib)_5_-OMe [81] that existed in a helix with *n* to *n-3* H-bonding and an angle of ~100 degrees between α-carbons, similar to the 3.6_13/10_ proposed herein based on high-resolution crystal structures. This helix was very similar to one proposed earlier by Némethy et al. in 1967 [9] based on analytical analysis of helical parameters, except the α_II_-helix had no hydrogen bonding. An intermediary helix between the α_I_- and α_II_-helix shown in the paper (**Fig 3**), however, had “hydrogen bonding arrangements of these residues is intermediate between those of the α_I_-helix and the (3_10_)_I_-helix, while the helical parameters *n* and *h* are still those for the α_I_-helix.” [9] It is ironic that some 47-years later, the “Némethy”-helix (3.6_13/10_-helix) has been validated as dominant in protein structures, and by a group including one of the students (GRM) whom Prof. George Némethy tutored at Rockefeller University.

**Fig 19:**
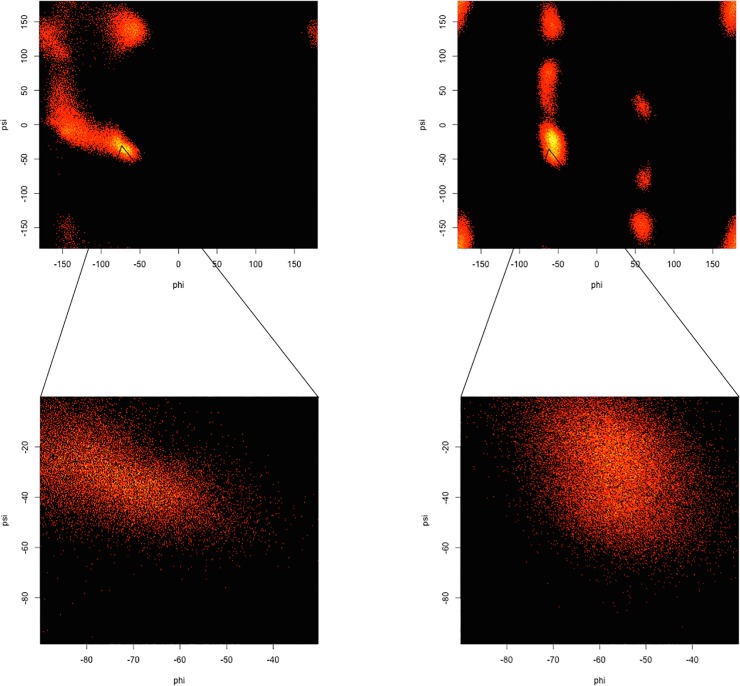
The whole (top) and expanded (lower) Ramachandran plots of 12-residue oligo-Ala (left sides) and oligo-Aib (right sides) from AMOEBA replica-exchange MD simulation. The 3_10_-region in Aib is heavily occupied while in alanine it’s almost absent.

**Fig 20:**
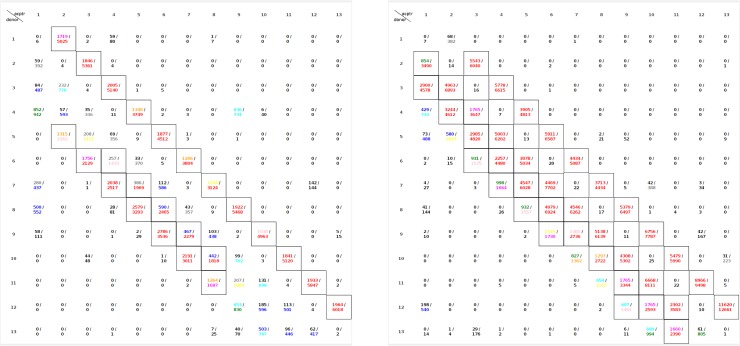
The hydrogen-bonding matrix of 12-residue capped oligo-Ala (left) and oligo-Aib (right) from AMOEBA replica-exchange MD simulation. The acceptor+1(*i*->*i+2*) and acceptor+2(*i*->*i+3*), indicating 3_10_ helix) in oligo-Aib is more heavily occupied than in oligo-Ala.

While the analysis of Hodgkin et al. was very thorough, it depended on an early version of AMBER. In order to test this hypothesis with a more precise force field, MD simulations in water of 12-residue capped oligoalanine and oligo-α-aminoisobutyric acid were preformed with AMOEBA. The Ramachandran plots from the 10-nsec replica-exchange MD simulations are compared (**Fig 19**). The hydrogen-bonding matrices for oligo-Ala and oligo-Aib from the MD simulations (**Fig 20**) show a significant increase in 3_10_-like helices in the oligo-Aib peptide. Thus, introduction of Aib residues enhances the preference for 3_10_-like helices and limits the relevance of data from such peptides to protein helices.

### Role of three-atom-centered hydrogen bonds

Hydrogen bonds are dominated by electrostatic forces, and are consequently weak, directional, promiscuous, with compensatory features not accurately represented by a single linear potential. Jeffrey and Saenger forecast in 1991, “In future high-resolution crystal structure studies of proteins, hydrogen bonding has to be analyzed in terms of two-centered and three-centered interactions. It should be stressed that three-centered bonds may be especially important in protein dynamics because they can be regarded as transitional intermediates from one two-centered bond to another.” [6]

Simultaneous, shared hydrogen bonds are conceptually equivalent to the networked hydrogen bonds in proteins as recognized by Stickle et al. [41], “. . .almost all central residues of helices are within networks because helix geometry is commensurate with both i → i+4 and i → i+3 hydrogen bonds simultaneously. . .” and “. . .it is likely that the existence of multiple determined hydrogen bonds enhances the elasticity of an α-helix”

Mixed DFT/AM1 quantum calculation on oligoalanine helices by Wieczorek and Dannenberg [82] found “hybrid” 3_10_/α structures in which “C = O forms both 3_10_- and α-helical H-bonds.” Bour et al. found average torsional angles of ϕ = -63.3 and ψ = -42.1 for capped alanine nonapeptides using HF/DFT with COSMO solvation [71]. Thus, a consensus from both experimental and theoretical methods strongly supports three-atom-centered (bifurcated) hydrogen bonds as found in the Némethy, N-, 3.6_13/10_-helix first proposed in 1967 [9].

Nevertheless, the classical α-helical and 3_10_-helical models of linear hydrogen bonds still dominate structural literature on proteins, and strongly influence conceptual views of protein dynamics. Estimates of the strength of bifurcated hydrogen bonds by quantum mechanics in model systems indicate negative cooperativity regarding the calculated interactive energy per hydrogen bond [83,84]. In effect, donors and acceptors become less avid hydrogen-bonding partners experimentally once they are already participate in a hydrogen bond [85], but the total stabilization of the two hydrogen bonds in the three-centered hydrogen bond is greater than a single two-centered hydrogen bond. The actual energetics of bifurcated H-bonds in proteins are still under investigation [86].

### Monopole vs. Multipole Electrostatics

More than three decades of literature clearly cite the inability of monopole electrostatics to reproduce the molecular electrostatic potential calculated by *ab initio* quantum mechanics. For example, Williams showed that inclusion of dipole and quadrupole terms were required to accurately mimic *ab initio* electrostatic potentials of small molecules, such as water [20,21]. Stone has argued since the 1980s that distributed multipole analysis (DMA) derives a much more accurate description of the electrostatic potential surrounding a molecule in terms of charges, dipoles, and quadrupoles compared with a calculated wave function [19,22,87]. Ren and Ponder have shown that inclusion of polarizability and multipole electrostatics, derived with DMA, was necessary to reproduce the colligative properties of bulk water and the local geometry of water clusters using a single consistent set of parameters [18]. Schneiders et al. have presented a convincing case for the use of the polarizable AMOEBA force field in crystallographic refinement [28]. In preliminary studies comparing molecular dynamics simulations with explicit solvent of high- resolution crystal structures using AMOEBA versus OPLS-AA and CHARMM, Kuster [37] found that AMOEBA preserved the experimental helical length of crambin 7–18 (PDB = 1CRN, resolution = 0.945 Å) while OPLS-AA and CHARMM both shortened the helix approximately 0.5 Å to a length corresponding to an ideal α-helix. These results support the view that monopole electrostatics used in some low-resolution structural refinements introduced α-helical bias.

### Dipoles of helices and external fields

**Fig 21:**
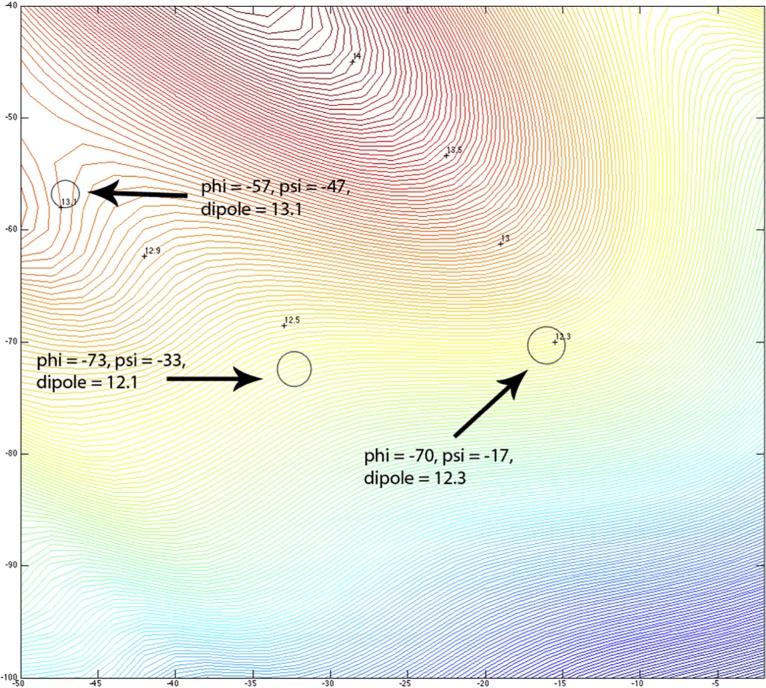
Contour plots of oligo-Ala dipole moment. The dipole moment magnitudes of the classic α-helix, 3_10_-helix and Nemethy-, N-, 3.6_13/10_-helix are marked with circles.

In order to determine the dipole change during the transition from 3_10_-helix to α-helix, the dipole moments from QM *in vacuo* in the α-helical region was plotted (**Fig 21**). The reorientation of the amide bond seen in the experimental Némethy-, N- or 3.6_13/10_-helix might be expected to impact the dipole moment of the capped trialanine helix. Hol [88] and others [89,90] have considered the roles of helix dipoles in protein function. Ripoll et al. have shown that the dipole moments associated with α-helical conformations showed alignment with the local electrostatic field in a large set of native proteins [68]. The QM-calculated dipole for the different Ac−Ala−Ala−Ala−NMe helices in this study varied from 12.1 Debye for the Nemethy-, N-, 3.6_13/10_- helix to 13.1 Debye for the α-helix (**Fig 21**), hardly a significant change.

Calculation of the dipole moments for Ac−(Ala)_12_−NHMe, however, using a web-based server for Adaptive Poisson-Boltzman Solver [65] or with the AMOEBA charges gave very significant changes in dipole moments depending on helical conformation (**Table 6**). This implies another parameter for optimization in protein folding, orientation of the peptide-bond dipoles in response to the local electrostatic field that is consistent with the lack of linear groups observed in experimental protein structures [32]. Choudhary and Raines have found that amino acids, which adopt the main chain dihedral angles of an α-helix, display dramatic pyramidalization of carbon (Ci′) toward the oxygen (Oi−1) leading them to conclude that Oi−1 and Ci′ are linked by a partial covalent bond in α-helices [61]. This potentially significant quantum interaction has not been considered in this manuscript. Further implications of the reorientation of the amide bond remain to be explored. The current second-generation force field AMOEBA, however, appears adequately parameterized to both reproduce this phenomena, and to allow exploration of its impact on protein structures.

## Conclusions

The structural and energetics of the helical region of proteins has been examined by X-ray and neutron crystallography, molecular dynamics simulations, monopole and second-generation force fields that include multipole electrostatics and polarizability as well as quantum mechanics (focused primarily on the (φ = -100 → 40, ψ = -50 → 0) region of backbone torsional space). From a detailed examination of X-ray crystallography data in the PDB, systematic differences in torsion-angle distributions between high- and low-resolution experimental data were observed. A single Gaussian-like peak was observed in the high-resolution data, challenging the classical α-and 3_10_-helical hypotheses that were based on linear hydrogen bonding. Since low-resolution X-ray crystallography and NMR data have utilized force-field constraints in refinement (until very recently, with inaccurate monopole electrostatics), serious concerns are raised regarding the accuracy of some data, particularly models derived for low-resolution diffraction data, in the Protein Data Bank.

The torsional distribution of oligoalanine in AMOEBA09 MD simulations in water showed a single peak at φ = -72.78, ψ = -33.43, once again challenging the two canonical helices hypothesis resulting from monopole electrostatics and linear hydrogen bonding. The role of water in protein structure and dynamics cannot be overemphasized [91,92], and MD simulations, assuming an accurate force field, are a viable way to explore solvation. Overall, protein helices are dynamic and flexible in responding to their environments; only in unusual circumstances could they be considered “linear groups”. Rather, carbonyl and amide bond groups optimize the electrostatic interaction of their individual dipoles with the local, surrounding fields [68] which is generally possible to accomplish without requiring significant changes in the position or orientations of the peptide Cα-Cβ side chain vectors that impact packing. This is consistent with the variation in backbone torsional parameters seen with the crambin helix (**Table 4**).

In order to reconcile the experimental and theoretical data with the geometrical requirements of molecular orbital theory and a modern treatment of electrostatics, it was necessary to introduce slight adjustments of the backbone torsional angles associated with protein helices while still maintaining the overall α-helical parameters (rise-per-residue and pitch). Surprisingly, this crankshaft-like rotation of the amide bond does not move the Cα and Cβ atoms from their positions in the Pauling α-helix, a fact that may explain ongoing structural support for linear hydrogen bonding. It is important to recognize that this crankshaft rotation does, however, accommodate three-centered amide hydrogen bonding (typically with a shorter and stronger *i* → *i+4* compared to the longer and weaker *i* → *i+3* hydrogen bond). Thus, minor refinement of helical backbone torsional angles to those of the consensus experimental high-resolution crystal structure (φ = -62, ψ = -43) is recommended as being most consistent with all available experimental and theoretical considerations. We designate this the Némethy-, or N-helix (3.6_13/10_) having the same helical parameters (rise-per-residue and pitch) as the classical α-helix, and modified backbone torsional angles to accommodate three-centered hydrogen bonds.

The resulting perspective reconciles requirements of carbonyl molecular orbitals, the enhanced strength and entropic benefits of three-centered hydrogen bonds, second-generation force fields with multipole electrostatics and polarizability with high-resolution crystal structures of protein helices. This self-consistent view of protein helices abandons the linear hydrogen bond without modifying the overall (crystallographically observable) heavy-atom positions (with the exception of the oxygen of the amide carbonyl) and helical parameters of the classic α-helix of Pauling, Corey and Branson [1].
